# Supplementary figures and images for: A new polymodal gating model of the proton-activated chloride channel
Source: PLoS Biol. 2023 Sep 15;21(9):e3002309. doi: 10.1371/journal.pbio.3002309 (PMC10529583; doi:10.1371/journal.pbio.3002309)

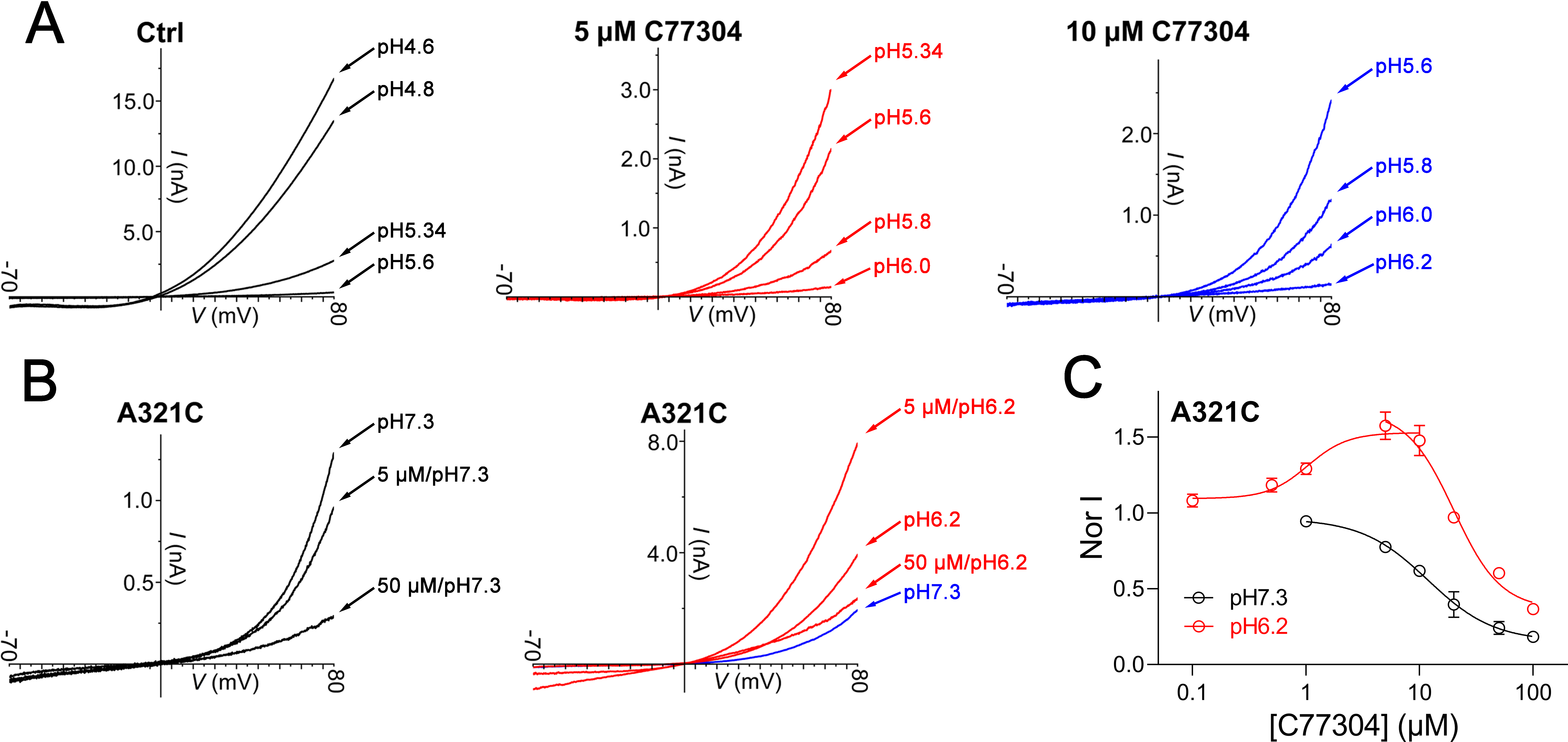

Supplement: S2 Fig — (A) Representative PAC currents elicited by different acidic pH solutions in the absence (left) and presence of 5 μM C77304 (middle) or 10 μM C77304 (right). Note that the C77304 treatment changed the threshold and saturating activation pHs. Currents were recorded with ramp depolarizations from −70 mV to +80 mV (n = 7–12 cells per condition). (B) Representative current traces showing C77304 monotonically inhibits (left) or bidirectionally modulates (activating and inhibiting; right) PAC/A321C mutant channels at pH 7.3 and pH 6.2, respectively (n = 5). (C) Concentration–response relationships of C77304 acting on PAC/A321C mutant channel, with the curves being Sigmoidal or bell–shaped at pH 7.3 and pH 6.2, respectively. The EC50 for C77304 activating PAC currents at pH 6.2 was 1.0 ± 0.4 μM, and the IC50 for inhibition were 11.8 ± 1.9 μM and 20.0 ± 2.9 μM at pH 7.3 and pH 6.2, respectively (n = 5). The data underlying the graphs shown in the figure can be found in S1 Data. (TIF) [file pbio.3002309.s003.tif]

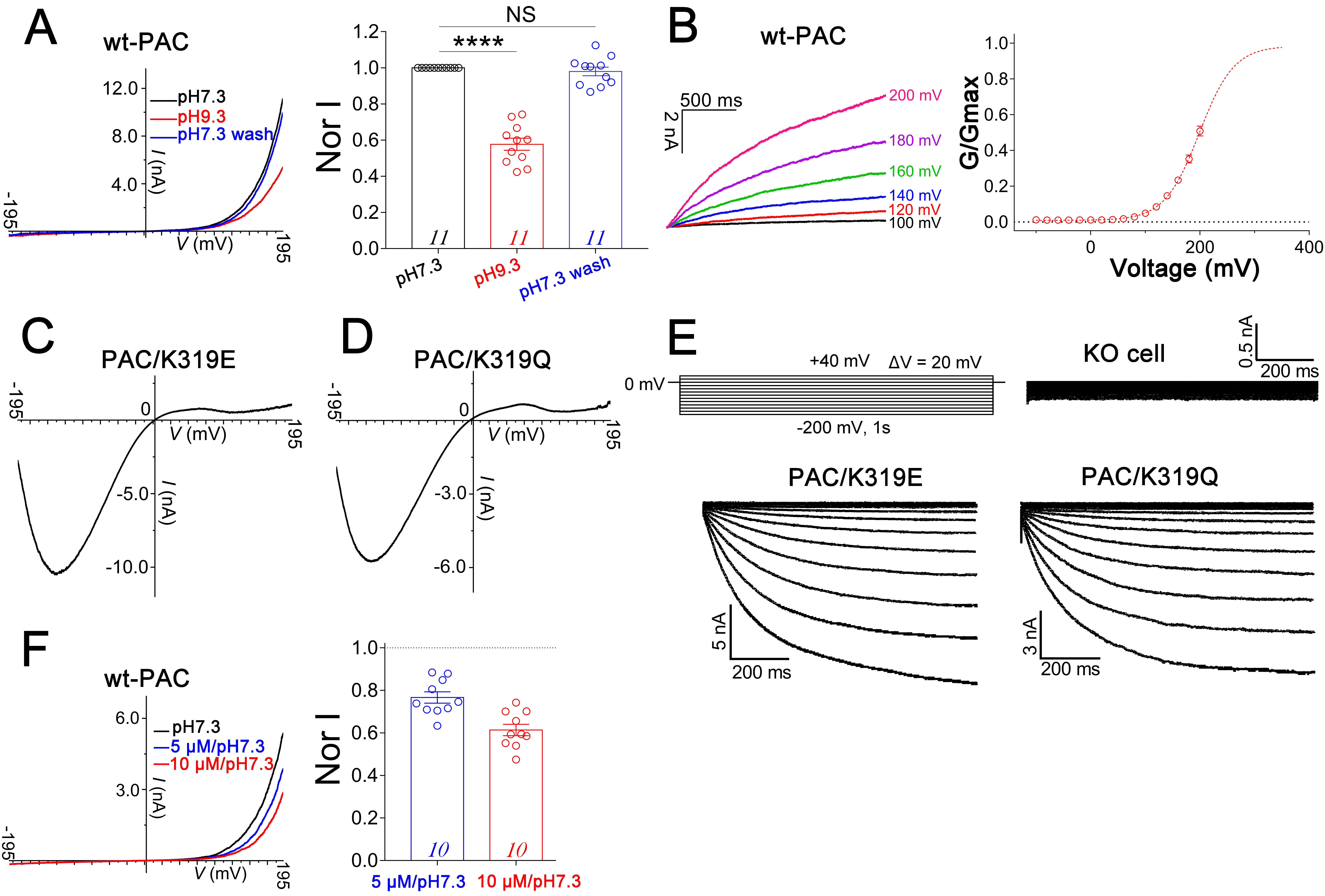

Supplement: S3 Fig — (A) Representative current traces (left) and summary of normalized currents (Nor I, right) showing alkali treatment reversibly reduced the strong depolarization–activated PAC currents (n = 11); statistics were evaluated by paired t tests (****, p < 0.0001; NS, not significant). (B) Representative current traces of wt–PAC channels elicited by step depolarizations from −100 mV to +200 mV (in 20 mV increment) at pH 7.3 (left panel) and the normalized conductance vs. voltage relationship (right panel). The half maximal activation voltage (V1/2) and the slope factor (K) were determined as 198.7 ± 13.4 mV and 31.7 ± 3.4 mV, respectively (n = 9). (C, D) Representative current traces of PAC/K319E (C) and PAC/K319Q (D) mutant channels elicited by ramp depolarizations from −195 to +195 mV at pH 7.3 (n = 7). (E) Representative current traces of PAC/K319E and PAC/K319Q mutant channels activated by depolarizing/hyperpolarizing voltage steps from +40 to −200 mV (in increments of 20 mV) at pH 7.3. The HEK293T/PAC–/–(KO) cell (lacking any PAC channels) was used as the control (n = 10–15). (F) Representative traces (left) and summary of normalized currents (right) showing that C77304, in a concentration–dependent manner, inhibited the strong depolarization–activated PAC currents at pH 7.3 (n = 10). The data underlying the graphs shown in the figure can be found in S1 Data. (TIF) [file pbio.3002309.s004.tif]

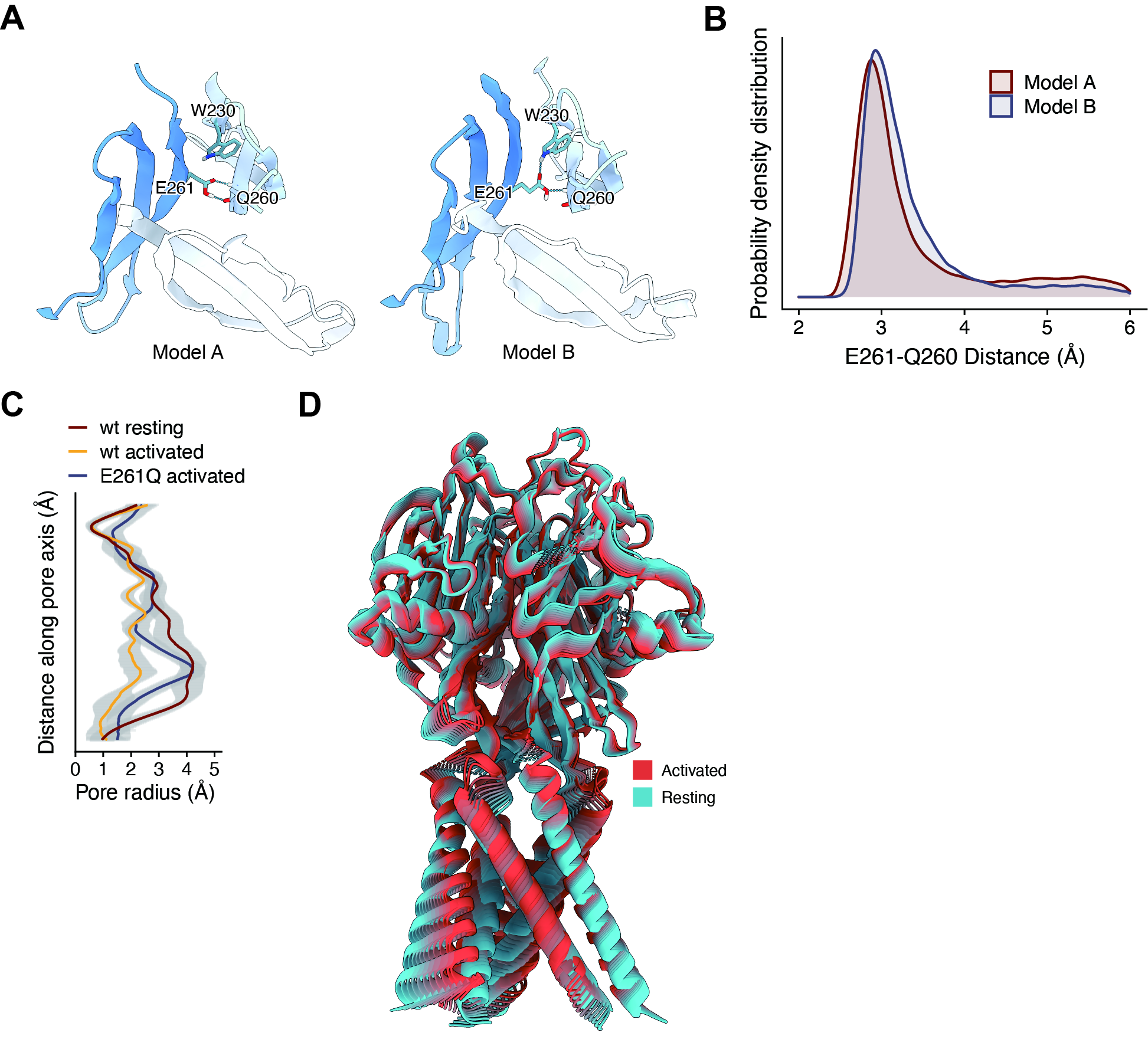

Supplement: S8 Fig — (A) The interactions between Q260 and the E261–W230 cluster on a neighboring chain in the activated state. The different orientations of E261 lead to models A (left) and B (right). (B) The probability density distributions of the distance between the carboxyl oxygens on E261 and on Q260 in the 2 models. (C) The pore sizes of the wild–type PAC channel in the resting and activated states and the E261Q mutant PAC channel in the activated state. The pore sizes are sampled from the MD trajectory of the system containing only ECD. (D) The predicted structural transformation from resting state to activated state were made using Morph. The data underlying the graphs shown in the figure can be found in S1 Data. (TIF) [file pbio.3002309.s009.tif]

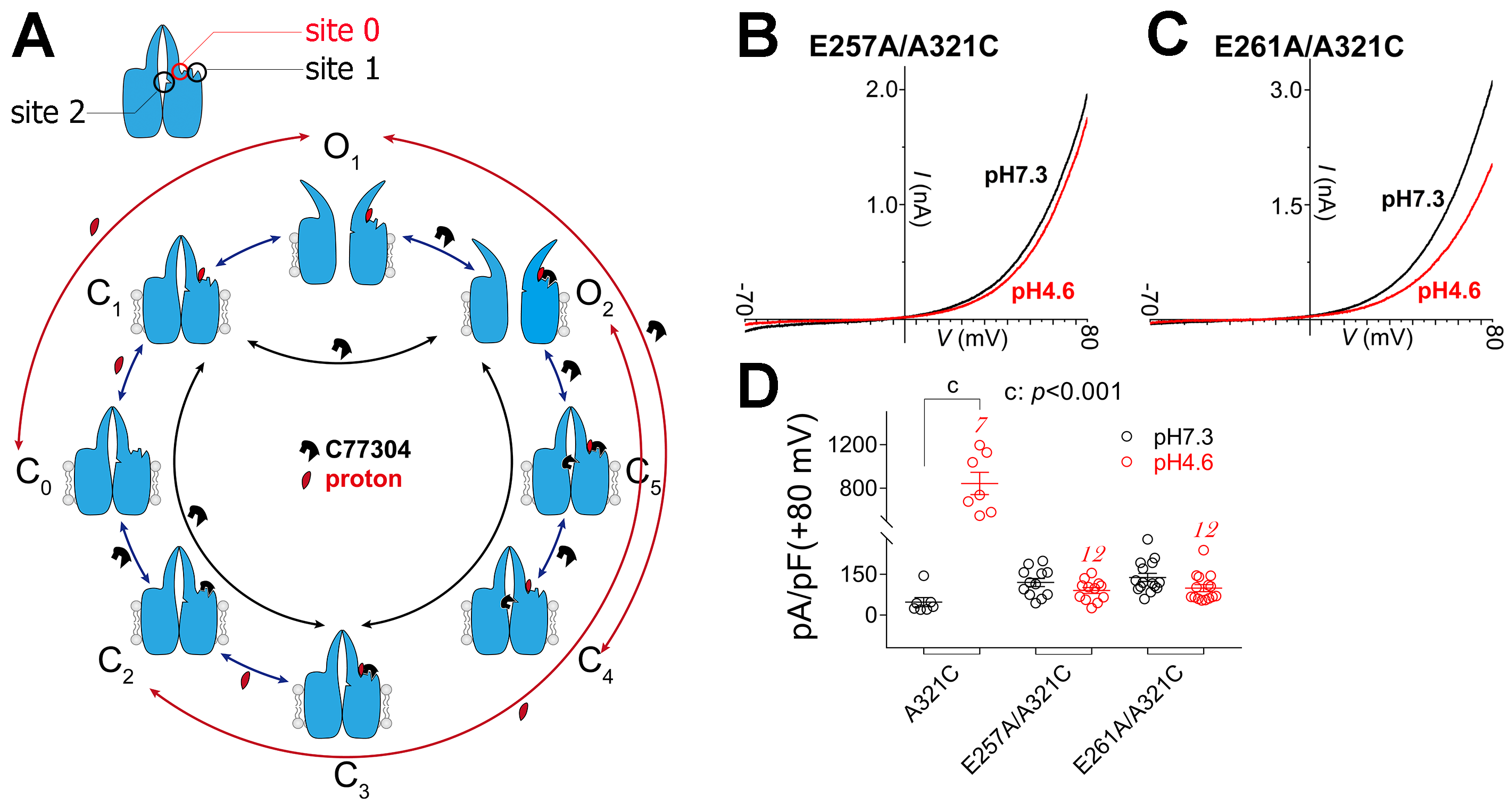

Supplement: S9 Fig — (A) Proposed model illustrating the binding/unbinding of protons and/or C77304 with PAC channel and the resulting state transitions. “C0–C5” and “O1–O2” represent different closed and open states, respectively. Site 0, proton binding; site 1, activation site; site 2, inhibition site. (B, C) Representative current traces demonstrating that the PAC–E257A/A321C (B) and PAC–E261A/A321C (C) double mutant channels are not activated by protons but exhibit considerable basal opening at pH 7.3 (n = 12). Currents were recorded with a voltage ramp from −70 mV to +80 mV. (D) Summary analysis of the proton–activated currents of PAC/A321C, PAC–E257A/A321C, and PAC–E261A/A321C mutant channels, significant differences between the pH 7.3 and pH 4.6 conditions were assessed using a paired t test; p value as indicated in the panel and n values as indicated in each bar. The data underlying the graphs shown in the figure can be found in S1 Data. (TIF) [file pbio.3002309.s010.tif]

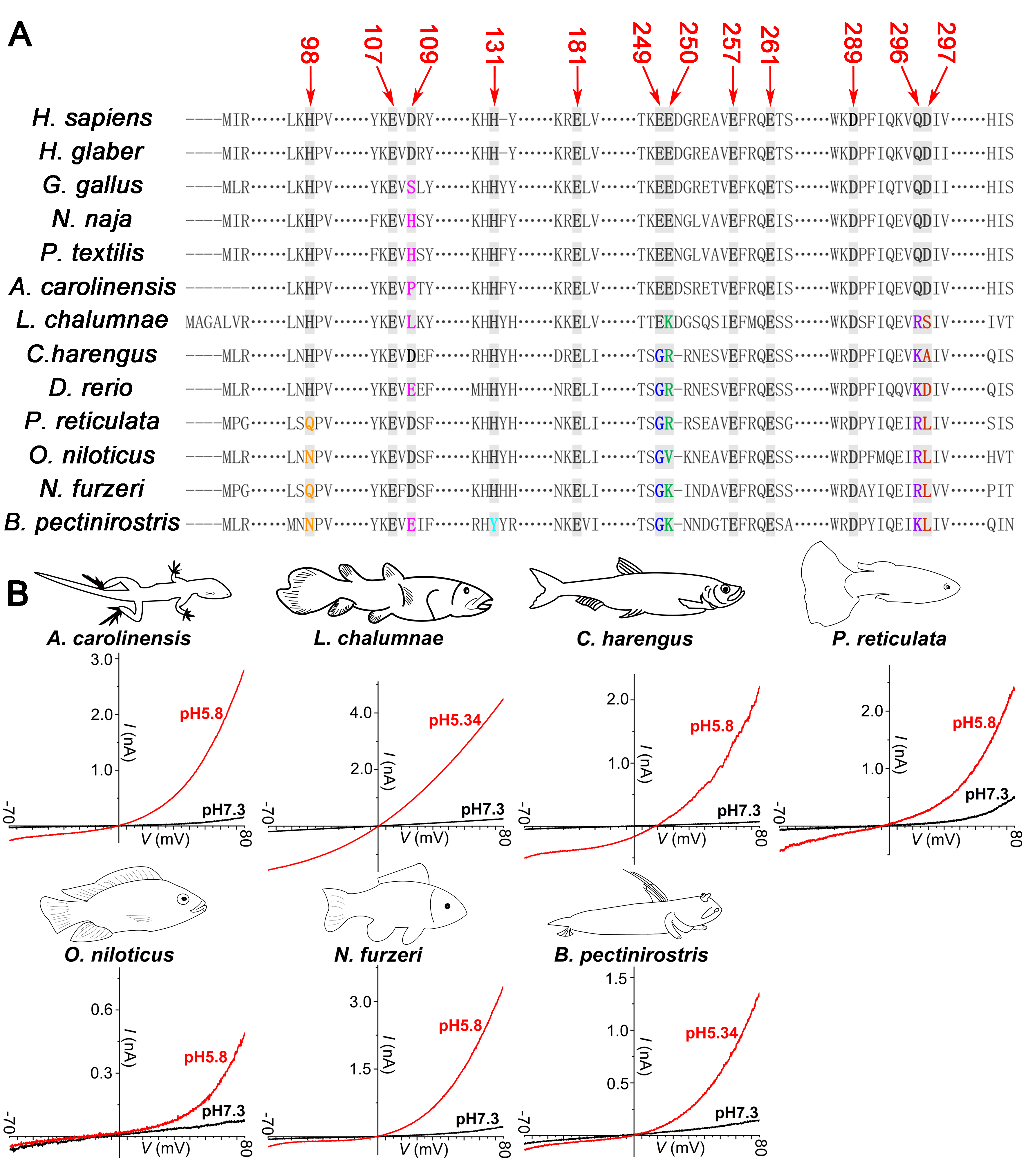

Supplement: S10 Fig — (A) Sequence alignment of PAC channels from 13 different species as indicated, showing that residues E181, E257, and E261, but not the other gating–related residues are conserved across species (in human PAC numbering). In PAC channels from P. reticulata, O. niloticus, and N. furzeri, the proposed proton sensor H98 is mutated to glutamine or asparagine; in PAC channel from B. pectinirostris, the proposed proton sensors H98 and H131 are mutated to asparagine and tyrosine, respectively; in PAC channels from C. harengus, P. reticulata, O. niloticus, N. furzeri, and B. pectinirostris, the E249 and D297 analogous sites mutation are expected to abolish the proposed E107–E249 and E250–D297 carboxy–carboxylate interactions in the activated state; the D109, E250, and Q296 residues which were proposed to form the H98 binding pocket are also varied among orthologous PAC channels from lots of species. (B) Typical proton–activated currents of PAC channels from A. carolinensis, L. chalumnae, C. harengus, P. reticulata, O. niloticus, N. furzeri, and B. pectinirostris (n = 4–9). (TIF) [file pbio.3002309.s011.tif]
