# Supplementary material for: A new polymodal gating model of the proton-activated chloride channel
Source: PLoS Biol. 2023 Sep 15;21(9):e3002309. doi: 10.1371/journal.pbio.3002309 (PMC10529583; doi:10.1371/journal.pbio.3002309)
Supplement: Attachment — Submitted filename: 0401_PAC MS_Point-By-Point Response Letter.doc [file pbio.3002309.s015.doc]

**point-by-point response to Reviewer #1**

**Reviewer #1:**

In my opinion this is a very interesting paper, reporting novel results on the activation mechanism of PAC. The authors use a clever approach, namely a bidirectionaly pharmacological modulator to investigate the gating mechanism of the channel. Overall the paper is well written, uses appropriate methods (scanning mutation analysis, molecular dynamics simulations and cross-species analysis) and statistics that are clearly described, to reveal novel findings concerning proton gating-independent voltage gating.

I am a bit concerned however about the MD simulation. The authors state that in order to safe computational time only the extracellular domain was simulated. Wouldn't the transmembrane domain influence gating? How did the authors make sure that the results of the gating model are not biased, due to this (perhaps understandable) omission?

Thus, I recommend minor revision of the paper, addressing the above question.

**Response:**

We greatly appreciate the reviewer’s valuable comments on our manuscript.

The simulations with the ECD-only segment do not imply that the transmembrane domain does not affect the extracellular domain. In our previous MD simulations, we retained the linker at the ECD-TMD interface. We noticed that the interface did not reflect a significant conformational change. Of course, when the system undergoes a transition between states, e.g., from resting to activated, changes in the ECD are likely transmitted to the TMD through this linker. Therefore, using the ECD-only segment for the simulations of specific functional state seems reasonable. It is important to note that such a protocol has also often been used in the literature, for example, in the study of how the ECD of glutamate receptors affects its gating of the TMD. Please see, for example, Benoît Roux’s paper in Nature Structural & Molecular Biology, 8, 283, 2011.

To further confirm the effect of TMD, atomistic MD simulations of the full-length protein (ECD+TMD) were performed as suggested by the reviewer. In the simulations, the TMD segment of the protein was properly embedded in the fully hydrated POPC lipid bilayers. The setup of the simulations was kept consistent with previous ones and underwent careful energy minimization and equilibration, after which the trajectory lasted 200-400 ns. We accumulated three parallel trajectories for the resting state and five parallel trajectories for the activated state, respectively, to improve sampling. Based on the full-length MD simulations, we found that our proposed gating mechanism is still supported, consistent with the results obtained from the ECD-only simulations. The residue interactions and the gating model provided in the manuscript have been updated (revised Fig. 7 and revised Supplementary Fig. 8).

**point-by-point response to Reviewer #2**

**Reviewer #2:**

Zhao et al. proposed a polymodal gating model for the proton-activated chloride channel in this work. They characterize C77304 as a probe of PAC acting on residues near the proton binding site. The authors proposed that C77304 can simultaneously activate and inhibit PAC activity by binding to two places. Furthermore, they suggest that PAC can transit to the conducting state by an intrinsic gating mechanism independent of protonation. The authors found outward-facing E181, E257, and E261 residues responsible for proton sensitivity and that the C77304 binding site enclosed these residues.

The evidence presented in this work seems important to understand the gating mechanism of this physiologically relevant chloride channel—however, several problems undermine the enthusiasm for this manuscript.

**Response:**

Thanks for your kind review of our manuscript. We have carefully revised the manuscript according to these valuable comments, please see below for details.

**Point 1:** An essential part missing in their discussion is how protonation of these residues induces PAC pore opening.

**Response:**

In our proposed gating mechanism, the ECD experiences protonation and tightens inter-subunit packing. As shown in the revised Supplementary Fig. 8D, the conformation changes of ECD are transmitted to the TMD region, causing the outward rotation of the TMD segment. Multiple amino acids in the ECD are involved in the process, such as E261, E257 and E181, as further described below.

Residue E261, as a proton receptor, shifts from intra-subunit interactions in the resting state to inter-subunit interactions in the activated state. The hydrogen bond network is also altered during the protonation of residue E257, increasing the contacts between subunits. The increased inter-subunit interactions result in tighter subunit binding between ECDs. In this process, residue E181 always locks the conformation outside of the ECD through the hydrogen bonding network and may play a key role in the conformational transfer process. Such information has been added to the revised MS (Page 10, Lines 42-44; Page 11, Lines 1).

**Point 2:** The data description is hard to understand. The authors did not give clear reasons for each of their experiments, and their figures are packed with data. For example, Figure 2 has 16 panels. The data in Figure 2 is fundamental and can be broken into 2 or 3 Figures so readers can better appreciate it. It may be better to focus on finding the residues responsible for proton sensitivity.

**Response:**

Thanks for these valuable comments, we have revised the text and figures following these instructions:

1) we have carefully revised the text to improve the manuscript’s clarity in this revision. In particular, we have added reasons for conducting the experiments where appropriate to make the logic of the study and each experiment clearer;

2) we have broken Figure 2 into two separate figures, the revised Fig. 2 and Fig. 3, which focus on illustrating the state-dependent action of C77304 and the proton gating-independent voltage gating of the PAC channel, respectively. We also added new data in revised Fig. 3 addressing the mechanism of the PAC channel’s proton gating-independent voltage gating (please see point 4 for additional details), following the suggestions from both reviewer #2 and reviewer #3;

3) Mutation analysis is commonly used to investigate the role of specific amino acid residues in protein function. In the case of proton-gated ion channels, such as the PAC channel and acid-sensing ion channels, the key proton sensing residues (glutamine, aspartate, and histidine) are mutated to alanine to eliminate the channels' proton binding capacity and investigate their role in proton sensing. However, it's difficult to determine whether the mutated residue is truly responsible for proton binding or if it's structurally important for channel function _(Matthew L. Rook et al., Coupling structure with function in acid-sensing ion channels: challenges in pursuit of proton sensors)_.

In our study, we found that the PAC channel possesses a unique voltage gating machinery that is independent of its proton gating. By assessing the effects of mutating the candidate proton sensing residues on the channel’s voltage-dependent gating, we were able to determine its possible structural role in channel gating. Specifically, mutating the proton sensing residue to alanine should abolish the channel’s proton gating but not fundamentally affect the voltage-dependent gating.

To provide a more comprehensive understanding on the gating mechanism of the PAC channel, we have incorporated the voltage gating-related data into our manuscript.

**Point 3:** Their model presented in Figure 2A to explain the data is poorly justified. Additionally, there is not a single fit of the model to the data. Such analysis is critical to support that model can recapitulate the bidirectional modulation of PAC. Hence, the authors need to show that their model fits the data. Furthermore, the authors present a more refined gating model in Figure 2P. However, as with model 2A, it is unclear if model 2P fits the data and how this model relates and rule-out model 2A.

**Response:**

Thanks for these valuable comments, we apologize for not making it clear.

1) The PAC channel is gated by protons, and we have defined the proton binding site (proton sensors) on the channel as site 0. In our previous manuscript, we characterized only the activation site (site 1) for C77304 binding in the PAC channel and did not investigate the inhibition site (site 2). We apologize for this omission, we have now collected new data showing that a distinct inhibition site 2 is located in the pore region of the PAC channel. Mutations at site 2 greatly reduce the inhibitory effect of C77304, while leaving its activation effect unaffected. Please find these new data in the revised Figs. 4G-H, and the revised Supplementary Figs. 5A-D. Text related to these data were added to the revised MS on Page 8, Lines 3-18. We can conclude that there exist a proton binding site (site 0), a C77304 binding site 1 (mediating the activation effect of the compound), and a C77304 binding site 2 (mediating the inhibition effect of the compound) in the PAC channel.

2) When we examine the occupation states of site 0, site 1, and site 2 by protons and/or C77304, there are theoretically eight combinations (ranging from no site being occupied to all three sites being occupied), as shown in Table 1 in this response letter. However, based on our experimental data addressing the state-dependent binding of C77304 to the PAC channel, we found that certain occupation states cannot exist. Specifically, the state in which only site 2 is occupied by C77304, as well as the state in which both site 1 and site 2 are occupied by C77304 but without proton binding to site 0 cannot exist because C77304 binding to site 2 requires the channel to be in an open state (these unachievable states are marked with red letters in Table 1). Therefore, taking into consideration of the pore states (close or opened), there are theoretically 12 possible combinations (marked with black and blue letters in Table 1).

It's worth noting that we were investigating the channel’s states transition driven by proton and/or C77304 binding, therefore, we only considered the sequential binding of proton and C77304 with the PAC channel. That is, we were not taking into account of proton dissociation from the C_4_ and C_5_ states (please see these two states in the revised Fig. 2A or Table 1 in this letter), which would result in the states that only site 2 or only site 1&site 2 were occupied.

The gating of the PAC channel involves two distinct events, namely proton sensor protonation and pore opening, which are allosterically coupled. This means that the binding of a proton to site 0 does not always lead to pore opening. The similar allosteric gating mechanism is also found in the coupling between the voltage sensor activation and pore opening in the voltage-gated Na^+^, K^+^, and Ca^2+^channels. However, the possibility of pore opening without proton sensor activation is extremely low.

The experimental data showed that the PAC channel is not activated by C77304 binding to site 1 alone. On the other hand, the channel remains closed (pore closed) as long as site 2 is occupied.

Consequently, these constraints further eliminated the four possible channel states marked with blue letters in Table 1. Thus, only 8 channel states are practically achievable (marked with black letters in Table 1).

Based on this analysis, we developed the model in the revised Figure 2A illustrating the channel’s states transition driven by protons and/or C77304 binding. This model fully recapitulates the bidirectional modulation of C77304 on the PAC channel, with C77304 binding to sites 1 and 2 with differing affinities (Figs. 1G, 1I, and 1J) and state-dependence (Figs. 2B-D) to regulate channel gating. Specifically: i) in weak acidic conditions, the closed-state channels are of high abundance, low-concentrations of C77304 preferably binding to site 1 to activate the closed channels and produce a dominant activation effect of the whole-cell current; whereas high-concentrations of C77304 would inhibit those open channels, as well as activate those closed channels and then inhibit them to (as site 2 is exposed, Fig. 1E), producing an overwhelming inhibition effect; ii) in strong acidic conditions, the closed-state channels are of low abundance, the overwhelming inhibition of C77304 on the open channels would mask its activation effect and result in an overall inhibition of the whole-cell current.

Table 1:

|  | site 0 (the proton binding site) | site 1 (the activation site) | site 2 (the inhibition site) | pore state | practically achievable (Yes/No) | defined channel state |
| --- | --- | --- | --- | --- | --- | --- |
| no site occupied | × | × | × | C | Yes | C_0_ |
|  | × | × | × | O | No |  |
| site 0 occupied | √ | × | × | C | Yes | C_1_ |
|  | √ | × | × | O | Yes | O_1_ |
| site 1 occupied | × | √ | × | C | Yes | C_2_ |
|  | × | √ | × | O | No |  |
| site 2 occupied | × | × | √ | C | No |  |
|  | × | × | √ | O | No |  |
| site 0 & site 1 occupied | √ | √ | × | C | Yes | C_3_ |
|  | √ | √ | × | O | Yes | O_2_ |
| site 0 & site 2 occupied | √ | × | √ | C | Yes | C_4_ |
|  | √ | × | √ | O | No |  |
| site 1 & site 2 occupied | × | √ | √ | C | No |  |
|  | × | √ | √ | O | No |  |
| all 3 sites occupied | √ | √ | √ | C | Yes | C_5_ |
|  | √ | √ | √ | O | No |  |

Note: √: site occupied; ×: site unoccupied; C: pore closed; O: pore opened; C_0_, C_1_, C_2_, C_3_, C_4_, and C_5_ represent different closed states of the PAC channel, and O_1_ and O_2_ represent two open states.

We have incorporated this table into the **Supplementary information (Supplementary Table 1)** to clarify the elucidation of these channel states, text illustrating this table was also included.

3) We utilized two different models in our study. The first model, depicted in the revised Fig. 2A, was employed to examine the channel state transition prompted by proton/compound binding. The second model, shown in the revised Fig. 3N (the previous Fig. 2P), was a simplified equation demonstrating the connection between proton sensor activation (X→XH^+^, where X denotes the proton sensor, and XH^+^ signifies the activated proton sensor with a proton (H^+^) bound) and pore opening (C→O, where C and O indicate the closed and open pore, respectively). In this model, proton sensor activation and pore opening were allosterically coupled, which is typical in ligand-gated ion channels.

Our research uncovered that the depolarization voltage (V) could directly trigger PAC channel pore opening without the activation of its proton sensor (the model in revised Fig. 3N). We have added new data illustrating the voltage-gating mechanism of the PAC channel following the reviewer’s suggestions (please see point 4 below for details).

**Point 4:** The idea that PAC has an ion flux gating mechanism was not tested. The fact that the K319E mutant behaves so differently to WT is not enough to invoke a role of permeant ion in gating. Similar changes in ionic selectivity have been reported in TMEM16A but were attributed to differences in pore electrostatic potential (Nguyen et al., <https://doi.org/10.3390/ijms22042209>).

**Response:**

Thanks for bringing this to our attention. Indeed, the ion selectivity could be attributed to either the size-dependent mechanism or the electrostatic field mechanism, as proposed for the TMEM16A channel _(Wenlei Ye et al.,_ [_https://doi.org/10.7554/eLife.45187_](https://doi.org/10.7554/eLife.45187)_)_. Millimolar concentrations of divalent cations changed the ion selectivity of the TMEM16F channel and the underlying mechanism is proposed as they changed the electrostatic field of the pore _(Nguyen et al.,_ [_https://doi.org/10.3390/ijms22042209_](https://doi.org/10.3390/ijms22042209)_; Wenlei Ye et al.,_ [_https://doi.org/10.7554/eLife.45187_](https://doi.org/10.7554/eLife.45187)_)_. For the PAC channel, Ruan Zheng et al., showed that its anion selectivity when activated by proton is determined by the K319 residue in the pore region, for which mutating K319 to E319 converts PAC to be a cation-selective channel _(Ruan Zheng et al., Nature 2020; DOI: 10.1038/s41586-020-2875-7)_.

In the present study, we are not discriminating the aforementioned two mechanisms (the size-dependent mechanism and the electrostatic field mechanism). Instead, we demonstrate that there is no endogenous built-in voltage sensor in the PAC channel, and that the rectification direction of the currents through the channel pore is determined by the flow direction of selectively conducted ions. These data provided evidence for an ion-flux gating model. We have designed new experiments addressing this issue and these data were incorportated into the revised MS:

1) We mutated the K319 residue in the PAC channel to E, Q, or H, which changes its charge (positively charged, negatively charged, or neutral) and thus the current direction through the channel pore. We found that both PAC/K319E and PAC/K319Q channels are activated by strong hyperpolarization, whereas the PAC/K319H channel could be activated by both strong depolarization and strong hyperpolarization, at the neutral pH7.3 (the revised Figs. 3H and 3K; the revised Supplementary Figs. 3B-D). To confirm that the voltage-activated PAC channel have similar ion selectivity as that of activated by protons, we first analyzed the ion selectivity of these channels by an ion substituting strategy. As shown in the revised Figs. 3G-I, the wt-PAC channel conducts outwardly-rectified Cl^-^ currents, and both the PAC/K319E and PAC/K319Q channels conduct inwardly-rectified Na^+^ currents, when activated by strong depolarization and strong hyperpolarization at neutral pH7.3, respectively. However, the PAC/K319H channel could conduct both inwardly-rectified Na^+^ currents at strong hyperpolarization and outwardly-rectified Cl^-^ currents at strong depolarization, at pH7.3 (the revised Fig. 3K-M). We also noticed that the wt-PAC showed certain degree of permability to SO_4_^2-^, and the PAC/K319H mutant channel showed certain degree of permability to both SO_4_^2-^ and NMDG^+^, when solely activated by transmembrane voltage at pH7.3.

**The following explanation addresses the deduction that the PAC channel is voltage-gated by the ion flux.**

2) if the voltage sensor in the PAC channel is made up of channel’s charged residues (a built-in voltage sensor), there are two possibilities:

2-a), Assuming that some charged residues, aside from K319, contribute to the voltage sensor in the PAC channel, then PAC/K319E and PAC/K319Q mutant channels harboring the intact voltage sensing machinery (like the wt-PAC channels) would be activated by strong depolarization, which contradicts the experimental data (the revised Figs. 3G-H, 3J, the revised Supplementary Figs. 3B-D).

2-b), if K319 contributes to the voltage sensor, the charge-reverse mutation (K319E) might overturn the voltage dependence of activation and render the channel activated by hyperpolarizations, whereas neutralizing it (K319Q) is expected to abolish the voltage gating. The PAC/K319H channel, however, would be activated either by strong depolarization or strong hyperpolarization, depending on the charge state of the introduced histidine residue, as determined by its pK_a_ value in its local biochemical environment and the external pH. Nevertheless, the PAC/K319H channel would not be activated by both depolarization and hyperpolarization in this scenario. As addressed earlier, the experimental data do not support the assumption that K319 contributes to the voltage sensor either (the revised Figs. 3H, 3K, 3L; the revised Supplementary Figs. 3C-D).

3) Consequently, our data support the contention that the PAC channel lacks an inherent voltage sensor. We propose that the ion-flux gating mechanism accounts for the proton gating-independent voltage gating of the PAC channel. Specifically, the conducting ion dictates the voltage activation of both the wild-type PAC channel and its mutants, similar to what has been observed in the K2P channel _(Schewe, M. et al., doi:10.1016/j.cell.2016.02.002)_. Our experimental data support this mechanism, for which the direction of current rectification is determined by the flow direction of selectively conducted ions, which means the flow ions gate the channel to increase its open probability:

3-a), the wt-PAC channel is selective for Cl^-^, strong depolarizations drive the Cl^-^ ions flow into the cell, which drives channel opening and produces outwardly rectified Cl^-^ currents;

3-b), the PAC/K319E and PAC/K319Q mutant channels are selective for Na^+^, strong hyperpolarizations drive the Na^+^ ions flow into the cell, which drives channel opening and produces inwardly rectified Na^+^ currents;

3-c), the PAC/K319H mutant channel is selective for both Cl^-^ and Na^+^, strong hyperpolarizations drive Na^+^ ions flow into the cell which drives channel opening and produces inwardly rectified Na^+^ currents, while strong depolarizations drive Cl^-^ ion flow into the cell, exhibiting outwardly rectified currents.

4) In this paper, we introduce the idea that the PAC channel is gated by voltage independent of its proton gating. We also used the voltage gating to investigate the potential structural role of the candidate proton sensor(s) on the channel. Although the present data support that the ion-flux gating underlies the voltage-dependent gating in the PAC channel, its detailed mechanisms are still to be clarified in future studies. It is likely that the residue constituting the selectivity filter in the wild-type and mutant PAC channels, the 319^th^ lysine(the wt-channel), glutamate(the K319E mutant), glutamine(the K319Q mutant), or histidine(the K319H mutant), acts as the interacting site of the conducting ion to transmit the voltage gating signals.

5) We have incorporated these new data into the revised Fig. 3, and texts related to this figure were also amended (Page 6, Lines 4-43).

**Point 5:** What is the explanation for the result: conservative mutant E181D and protonated-like E181Q mutant have both low proton affinity. Furthermore, E257R has a higher proton affinity, whereas E257Q has a lower proton affinity. How are these results conciliated with the idea that these residues are part of the proton sensing site? The analysis of E181A, E257A, and E261A mutants on the -/+195 mV range should also be included.

**Response:**

Thank you for raising this important point. The glutamate to glutamine mutation, which mimics the permanently protonated glutamate residue, is supposed to shift the pH_50_ of the PAC channel to the alkaline direction. However, as reported in published literature, it’s hard to predict this shift, as it depends on the local environment and secondary effects of mutations _(Chongyuan Wang et al., DOI: 10.1126/sciadv.abm3942; Matthew L ROOK et al., DOI: 10.1113/JP278707)_.

Here, we mutated the candidate proton sensors to alanine residues to eliminate the channel’s ability to bind protons, which would then completely abolish the proton gating. Currently, we do not have a good explanation for the acidic-direction pH_50_ shift caused by the E181Q mutation. The E257Q mutation shifted the pH_50_ to acid as well, which is consistent with a published study. _(Chongyuan Wang et al., DOI: 10.1126/sciadv.abm3942)_. We interpreted the E181D mutation causing a remarkable acidic-direction pH_50_ shift as “the side chain length might affect the channel’s proton gating as well”.

Regarding the E257-D289 coupling observed in the activated structure of the PAC channel, we propose that this interaction is *not* the primary driving force triggering the channel’s proton activation, but it may facilitate channel gating. We base this conclusion on the following evidence:

1) the E257R mutation significantly shifted the current-pH relationship to the alkaline direction, whereas the D289R mutation produced a large basal opening at pH7.3 without a pH_50_ shift (Fig. 5F, Supplementary Fig. 6C, Supplementary Table 2);

2) in contrast, the E257Q/D289N as well as the E257R/D289R double mutations caused a remarkable acidic-direction pH_50_ shift (Fig. 5F, Supplementary Fig. 6C, Supplementary Table 2);

3) the E257R/D289R mutations were predicted to destroy the proposed pairing to eliminate channel gating, however, this was not observed experimentally.

Based on a thorough mutation analysis of the candidate proton sensing residues in the human PAC channel (all glutamate, aspartate, and histidine residues), we conclude that the E181, E257, and E261 residues constitute the proton sensors. The mutational analysis of the analogous residues in PAC channels from other species (*H. glaber, P. textilis, N. naja, G. gallus, and D. rerio*) (cross-species analysis) also supports this conclusion. The conclusion that E257 forms the proton sensor in the PAC channel is also supported by another study published during the review process of our manuscript _(Xiang-Ying Xu et al., Identification of the acid-sensitive site critical for chloral hydrate activation of the proton-activated choloride channel; Journal of Neuroscience; 2023 Jan 25)_

4) the analysis of E181A, E257A, and E261A mutants on the -/+195 mV range was presented in the revised Figs. 5B, 5D, and 5E in this revision.

**Point 6:** The authors found that alkaline pHs decreased PAC activity. However, they concluded that these effects were due to membrane damage. It is unclear what kind of damage is caused by alkaline pH and if the membrane is damaged. From the records, it is not apparent a problem. Is the membrane leaky? The authors need to consider this observation in detail to rule it out as a direct effect of alkaline conditions on PAC.

**Response:**

Thanks for this valuable suggestion. To investigate the possible gating of the PAC channel by alkali, we tested if alkaline pHs reversibly reduce PAC currents, as follows:

1) the PAC currents rundown should be irreversible or slowly recoverable if the reason for the rundown is caused by membrane damage by alkali treatment, which is supposed to be irreversible or recoverable very slowly;

2) in contrast, PAC currents rundown should be fully and quickly recovered after a quick switch from pH9.3 to pH7.3 bath solution, if alkali conditions directly gates the PAC channel.

Our new data (see revised Supplementary Fig. 3A) fit the second assumption and thus support the idea that PAC channels might be gated by alkaline conditions. In fact, recent studies showed that OTOP1, another proton-gated ion channel, is also gated by alkaline conditions _(Bochuan Teng et al., DOI: 10.7554/eLife.77946; Lifeng Tian et al., DOI: 10.1038/s41467-022-35754-9)_.

These new data do not change the previous conclusion that the PAC channel is gated by strong depolarizing voltages independent of its proton gating, as the slope factors of the channels’ current-pH relationships at the pH ranges of 7.3-9.3 (where proton concentrations are very low) and <6.0 (where the PAC channel is typically gated by proton) are distinct (the revised Fig. 3F). The possible alkaline based gating of the strong-depolarization-activated PAC channel could be a future research interest.

**Point 7:** What do you mean by "thus enabling determination of their respective root pHs for mapping the residues comprising activation site 1" on page 5.

**Response:**

We apologize for the lack of clarity in our previous statement regarding "root pHs". The “root pHs” refer to the pHs near the threshold activation pH of the PAC channel, wherein most of the channels are in a resting state.

It’s worth noting that only at this pH range can we evaluate if the channel mutation affected the activation effect of C77304, thereby enabling us to determine if the mutated residue is critical for forming activation site 1, as the compound would solely inhibit the mutant channel’s currents at pHs more acidic than its root pHs (the revised Fig. 1G). For example, even if a mutant channel has an intact activation site (site 1), its currents would be monotonically inhibited but not activated by C77304 at pHs more acidic than the root pHs, leading to the false conclusion that the mutation eliminated compound binding to site 1.

**Point 8:** Is C77304 a competitive allosteric modulator?

**Response:**

Based on our data, we do not think that C77304 is an allosteric modulator competing with protons to activate the PAC channel. This conclusion is based on the fact that the compound alone is unable to activate the PAC channel at pH7.3, as shown in revised Fig. 2E. Instead, it might function by either enhancing the proton binding to the proton sensor (binding effect) or promoting the transition of the protonated channels to the open state (gating effect), both of which would result in the alkaline direction shift of the channels’ current-pH relationship (the revised Figs. 2F-G).

Regarding the inhibitory effect of C77304 on the PAC channel, we have added new data in this revision demonstrating that it binds to the inhibition site (site 2) located at the channel’s pore region (revised Figs. 4G-H and revised Supplementary Figs. 5A-D). The detailed mechanisms, allosterically hindering the pore opening or physically blocking the pore, however, require further investigation in future studies.

**Point 9:** Please include the pH_50_ vs voltage to support the statement on lines 40-43 of page 4;

**Response:**

Thanks for bringing this to our attention. We have included new data demonstrating the current-pH relationships at +40 mV and +80 mV are superimposed (the revised Fig. 2F). These data also support the contention that the channel binds with protons in a voltage-independent fashion.

**Point 10:** Reference is needed here on line 37, page 2;

**Response:**

We have added appropriate reference here (Page 2, Line 39 in this revised MS).

**point-by-point response to Reviewer #3**

**Reviewer #3:** Chloride currents activated by acidification of the extracellular pH (PAC) were first recorded in Sertoli cells in 2003; however, the underlying gene (TMEM206) was only identified in 2019. The molecular pharmacology and physiology of the channel is still in its infancy. Yet the channel could be an important therapeutic target in conditions in which extracellular pH may be pathologically lowered (e.g. ischaemic stroke, cancer etc.); thus, the elucidation of the mechanisms of pharmacological control of the channel could have implications for development of new therapies. The manuscript by Zhao et al. shows that C77304 has a biphasic effect on PAC channel activity. While I find this observation interesting and potentially important, I have some serious reservations about how some of the experiments have been conducted and interpreted. The text is also in some parts rather hard to read and it will require significant re-editing to increase clarity and amend grammatically imprecisions. This also extends to the figure legends and in general the presentation of information in the figures which is often not intuitive - this makes it difficult for the reader to follow easily the authors' scientific arguments.

**Response:**

Thanks for your kind review of our manuscript. We have carefully revised the manuscript according to these valuable comments, please see below for details.

Major comments include:
**Point 1:** Page 3, line 25. The authors are referring to screening of a compound library, but no reference to this work nor an account of how the screening was produced is presented in the text. This information is essential and should be part of this pharmacological paper.

**Response:**

Thank you for bringing this to our attention. We have added details of the screening: “Pharmacological agents interfering with the proton gating of the PAC channel are valuable molecular tools for dissecting its gating mechanism. We screened a compound library (from Selleck Chemicals LLC, Catalog No. L3600) for such agents using manual patch-clamp analysis. Compounds demonstrating >50% inhibition (at 10 µM) of PAC currents in HEK293T cells at pH4.6 were considered as positive ‘hits’ and tested further at a less acidic pH of 5.34 to evaluate any possible pH-dependence of inhibition”, on Page 3, Line 27-32, in this revision.

**Point 2:** For the PAC channel, at room temperature the pH_50_ (pH at which half maximal channel activation is achieved) is around pH 5.15 with no current observed >~pH 5.8 (as shown in Fig. 3 of the submitted manuscript and in a range of published papers). In Fig. 1B the authors find that when the channel is maximally or near maximally activated (i.e. pH < 5), C77304 acts as a low potency inhibitor. When the pH was brought to values that are below pH_50_, low concentrations of C77304 produced some activation, while higher doses still produced inhibition.

Note that the activation produced is still very small compared to the activation observed in response to acidic pH (<~5). Thus, I found Fig 1G misleading and I advise that the data in this diagram are normalised for the current measured at the most acidic pH tested (in the absence of the compound), as opposed to being normalised for the current measured at each individual pH in the absence of the compound. In this way, it will be more obvious to the reader that the activation produced by the compound is relatively modest. It should also be made more clear in the text that the data presented in Fig 1G represent the acute, short-lasting activating effect of the drug.

**Response:**

Thank you for these valuable suggestions.

1) Yes, the reviewer is right that the C77304 and proton-activated PAC channels at less acidic pHs (pH5.34, pH5.6, and pH5.8) accounted only a small proportion of the total channels available. As addressed in revised Fig. 2, C77304 potentiates PAC currents by either promoting proton binding to the channels (binding effect) or facilitating the transition of protonated channels (gating effect) to the open state. However, under weakly acidic conditions, the channel’s protonation probability is low due to low-concentrations of protons, even with the compound enhancing the proton binding with the channel. This limiting factor likely determines the apparent modest efficiency of the compound in activating PAC channels in weakly acidic conditions.

Following the reviewer’s suggestion, we repeated these experiments, and now we normalized the currents elicited by pH5.34 in the presence of different concentrations of C77304 (0 µM, 0.1 µM, 0.5 µM, 1 µM, 5 µM, 10 µM, 20 µM, 50 µM, and 100 µM) to that solely elicited by pH4.6 treatment (without C77304). Our results indicate that the compound and proton-activated PAC channels accounted for approximately 13% of the total available channels. In our previous experiments, we were missing the pH4.6-evoked currents when testing the effects of C77304 at pH5.34, pH5.6, and pH5.8, which prevented us from normalizing the currents to the maximum to re-graph the data. Therefore, we have incorporated these new data into the revised Fig. 1H and rewrittent the text as “The proton- and compound-activated channels, however, accounted for a small population of the total available channels (Fig. 1H)” (Page 4, Lines 8-10).

2) The green dot curve in the previous Fig. 1F was misleading, as it suggested some instability in the compound’s activation effect due to an apparent current run-down. Upon further examination, we found that it was caused by contamination from some leaky currents. We regret this mistake. We have repeated the experiments and incorporated the new data into revised Figs. 1E-F.

We would like to clarify that the activation effect of low-concentrations of C77304 on PAC currents is actually quite stable, as demonstrated by both revised Fig. 1F and Fig. 1K. However, high-concentrations of C77304 did transiently activate the channels before leading to a time-dependent inhibition (the revised Fig. 1E). This is in line with the idea that C77304 binds to the inhibition site (site 2) with strict state-dependency and lower affinity than its binding to the activation site (site 1). In this scenario, high-concentrations of C77304 would preferably bind to site 1 to activate the PAC channel, thereby exposing site 2, which is then occupied by the compound, causing current inhibition.

Throughout this paper, except for when testing the time-dependent effects of C77304 (revised Figs. 1E, 1F, 1K, 1L, and Fig. 2D), we measured and present the steady-state effect of the compound. In this revision, we also have characterized a separate inhibition site 2 located in the pore region of the PAC channel (please see our response to point 7).

**Point 3:** In addition, the interpretation offered by the authors that the different effects of the drug at different pH depend on different gating states of the channel seems incomplete, because the protonation state of C77304 will also vary (depending on the pKa of the molecule). This fact should be taken into account in the interpretation of the results.

**Response:**

Thank you for bringing this to our attention. Following the reviewer’s suggestion, we calculated the proportion of deprotonated C77304 molecules in solutions with pHs ranging from 4.6 to 7.4.

The pK_a_ of the carboxy group in C77304 was predicted to be 3.64 and 2.922 by Epik _(John C Shelley et al., Epik: a software program for pKa prediction and protonation state generation for drug-like molecules)_ and ChemDraw, respectively. The second pK_a_ for the NH group in C77304 does not come into play in this pH range (0% ionized up to pH10.0).

Based on the Henderson-Hasselbalch equation: *p*H = *p*K_a_ + log[A^-^]/[HA], where [A^-^] and [HA] represents the concentration of the deprotonated and neutralized molecule, respectively;

We can easily get the proportion of the deprotonated C77304 molecule ([A^-^]/[HA], denoted as ‘% ionized’) at each pH, as shown in the below figure:

Therefore, the ratio of the deprotonated C77304 moleclues at each pH as determined in this curve is:

|  | **% ionized** | |
| --- | --- | --- |
| **pH** | **Epik** | **ChemDraw** |
| 4.6 | 90.12 | 97.94 |
| 4.8 | 93.53 | 98.69 |
| 5.0 | 95.82 | 99.17 |
| 5.2 | 97.32 | 99.48 |
| 5.4 | 98.29 | 99.67 |
| 5.6 | 98.92 | 99.79 |
| 5.8 | 99.31 | 99.87 |
| 6.0 | 99.57 | 99.92 |
| 6.2 | 99.73 | 99.95 |
| 6.4 | 99.83 | 99.97 |
| 6.6 | 99.89 | 99.98 |
| 6.8 | 99.93 | 99.99 |
| 7.0 | 99.96 | 99.99 |
| 7.2 | 99.97 | 99.99 |
| 7.4 | 99.98 | 100.00 |

These data indicate that C77304 molecules are mostly deprotonated within a pH range of 4.6 to 7.4. Notably, the proportion of the deprotonated molecules remains stable within a pH range of 5.0 to 5.8 (with a maximum variation of 3.49%). However, at pH5.8, C77304 behaves as a bi-directional modulator, while it acts as an apparent channel inhibitor at pH5.0. It’s unlikely that this phenotype switch was caused by differing deprotonation state of C77304 at these two different pHs. We have included these data in the revised Supplementary Fig. 1E.

**Point 4:** Page 3, lines 32-33. "These data suggest that the C77304's effects might be driven by a competition between its activation and inhibition actions". It is not too unusual for a "gating modifier" compound to produce activation and inhibition. This may reflect binding to, and "conformational bias" towards different states of the channel. This point should be considered more fully in the Discussion.

**Response:**

Thank your for raising this important point. In this revision, we have characterized a separate inhibition site 2 located in the pore region of the PAC channel (please see our response to point 7 for details). We have revised the text to further clarify the rationale that C77304 exhibits a bifunctional modulation on the PAC channel by binding to two separate sites with different affinity and state dependency.

**Point 5:** Page 3, lines 35-37 and Fig 1F. The current measured at pH 5.6 was transiently activated by 50 uM C77304. When the cells were exposed to a solution supplemented with only 1 uM C77304, the current was activated and then again slowly declined (although the authors might have not waited long enough to achieve a true steady-state in this inhibition). Increasing the concentration of the compound to 50 uM at this point reduced current amplitude. This result is entirely predictable and the rationale for performing this experiment remains unclear.

**Response:**

Thank you for bringing this to our attention.

We confirmed that low-concentrations of C77304 have a stable activation effect on PAC currents in weak acidic conditions. In response to a concern raised, we have addressed this in point 2.

The logic for sequentially applying 1 µM and 50 µM C77304 is addressed below:

We observed that when 50 µM C77304 is applied to whole-cell PAC currents in weak acidic conditions (such as at pH5.6), a steady-state inhibition effect is always preceded by a transient activation of the currents (as seen in revised Fig. 1E). This suggests that the compound activates the channel before inhibiting it. We thus hypothesized that C77304 inhibition of the PAC channel requires the channel in the open state (the inhibition occurs following channel opening).

Therefore, if sufficient number of open-state channels are present on the membrane, 50 µM C77304 should directly inhibit the PAC currents without a transient activation effect. To further investigate, we conducted experiments to adjust the proportion of open-state PAC channels on the membrane by applying 1 µM C77304 first. This showed stable potentiation of the pH5.6-evoked currents (as seen in the revised Fig. 1F). Subsequent application of 50 µM C77304 directly inhibited the currents without a transient activation event (the revised Fig. 1F), which corroborates our hypothesis.

As we systematically examined the state-dependent inhibition of the PAC channel by C77304 in Fig. 2, we did not introduce the idea that C77304 inhibition requires the channel in the open state in the first section of the result part. Instead, we summarized the data from the revised Fig. 1A to Fig. 1F as “These data suggested that the proportion of resting-state PAC channels on the membrane, as well as the concentration of C77304, jointly determined the initial and steady-state effect of C77304 on the whole-cell PAC currents”, in this revised MS (Page 4, Lines 2-4).

**Point 6:** Page 4, line 2-6. The authors are not testing "binding and unbinding kinetics" of the compound. Measurements of an ionic current cannot constitute a direct measurement of binding. Here, the authors are simply observing the onset of the drug effect on the current at two different pH conditions. These conditions are associated with different (i) gating confirmations of the channel and (ii) protonation state of the molecule; therefore, the significance of this comparison is unclear.

**Response:**

Thank you for bringing this to our attention.

We agree with the reviewer’s point. The data in the revised Fig. 1K and 1L (the previous Fig. 1J and 1K) depicts the time-course of activation/inhibition of acutely perfused C77304 on PAC currents, as well as its wash-off upon bath solution perfusion. We acknowledge that this data does not accurately capture the "binding and unbinding" kinetics.

Regarding the protonation state of C77304, we have confirmed that it remains stable across different pHs we tested (as addressed in point 3). Nevertheless, we recognized that these experiments analyzed the binding of C77304 to PAC channels of two distinct conformational states at pH5.8 (when most channels are in the resting state) and pH4.6 (when most channels are in the opening state). Therefore, as the reviewer stated, the comparison of the time constants seem to be invalid. Therefore, we revised the text as follows: “The compound’s activation and inhibition effects remained stable after their fast onset upon acute perfusion (Figs. 1K-L). Additionally, it was observed that the activation effect was fully reversible while the inhibition effect was only partially reversible (Figs. 1K-L)” (Page 4, Lines 15-18).

**Point 7:** Page 4, lines 4-6. The authors claim that "These data strongly imply simultaneous binding of C77304 to the activation and inhibition sites of the channel with differing affinities". I strongly disagree with this conclusion, since I do not believe the authors have provided any evidence that there are separate binding sites for C77304 that may account for the transient activation and inhibition of the channel.

Thus, it appears that the basis of the conceptual model proposed in the section entitled "A model for C77304 bidirectionally modulating the PAC channel" are not sufficiently validated. The existence of the postulated sides 1, 2 and 0 is simply hypothetical.

**Response:**

Thank you for these valuable comments, we apologize for not making it clear.

1) The PAC channel is gated by protons, and we have defined the proton binding site (proton sensors) on the channel as site 0. In our previous manuscript, we characterized only the activation site (site 1) for C77304 binding in the PAC channel but did not investigate the inhibition site (site 2). We apologize for this omission, but we have now collected new data showing that a separate inhibition site (site 2) is located in the pore region of the PAC channel. Mutations within site 2 greatly reduce the inhibitory effect of C77304, while leaving its activation unaffected. Please find these new data in revised Figs. 4G-H and revised Supplementary Figs. 5A-D. Text related to these data were added to the revised MS on Page 8, Lines 3-18. Therefore, we conclude that there exists a proton binding site (site 0), the C77304 binding site 1 (mediating the activation effect of the compound), and the C77304 binding site 2 (mediating the inhibition effect of the compound) in the PAC channel.

2) When we examined the occupation states of sites 0, 1, and 2 by protons and/or C77304, there are theoretically eight combinations (ranging from no site being occupied to all three sites being occupied), as shown in Table 1 in this response letter. However, based on our experimental data addressing the state-dependent binding of C77304 to the PAC channel, we found that certain occupation states cannot exist. Specifically, the state in which only site 2 is occupied by C77304, as well as the state in which both sites 1 and 2 are occupied by C77304 but without proton binding to site 0 cannot exist because C77304 binding to site 2 requires the channel to be in an open state (these unachievable states were marked with red letters in Table 1). Therefore, taking into consideration the pore states (close or opened), there are theoretically 12 possible combinations (marked with black and blue letters in Table 1).

It's worth noting that we were investigating the channel’s states transition driven by proton and/or C77304 binding, therefore, we only considered the sequential binding of proton and C77304 with the PAC channel. That is, we were not considering proton dissociation from the C_4_ and C_5_ states (please see these two states in the revised Fig. 2A or Table 1 in this letter), which would result in the states that only site 2 or only site 1&site 2 were occupied.

The gating of the PAC channel involves two distinct events, namely proton sensor protonation and pore opening, which are allosterically coupled. This means that the binding of a proton to site 0 does not always lead to pore opening. The similar allosteric gating mechanism is also found in the coupling between the voltage sensor activation and pore opening in the voltage-gated Na^+^, K^+^, and Ca^2+^channels. However, the possibility of pore opening without proton sensor activation is extremely low.

The experimental data showed that the PAC channel is not activated by C77304 binding to site 1 alone. On the other hand, the channel remains closed (pore closed) as long as site 2 is occupied. Consequently, these constraints further eliminated the additional four possible channel states marked with blue letters in Table 1. Thus, only 8 channel states are practically achievable (marked with black letters in Table 1).

Based on this analysis, we developed a model in the revised Figure 2A illustrating the channel’s state transitions driven by protons and/or C77304 binding. This model fully recapitulates the bidirectional modulation of the PAC channel by C77304, with C77304 binding to sites 1 and 2 with differing affinities (Figs. 1G, 1I, and 1J) and state-dependencies (Figs. 2B-D) to regulate channel gating. Specifically: i) in weak acidic conditions, the closed-state channels are of high abundance, and so low-concentrations of C77304 preferably binding to site 1 to activate the closed channels and produce a dominant activation effect of the whole-cell current; whereas high-concentrations of C77304 would inhibit those open channels, as well as activate closed channels and then inhibit them (as site 2 is exposed; the revised Fig. 1E), producing a primarily inhibitory effect; ii) in strong acidic conditions, the closed-state channels are of low abundance, the overwhelming inhibition of C77304 on the open channels would mask its activation effect and result in an overall inhibition of the whole-cell current.

Table 1:

|  | site 0 (the proton binding site) | site 1 (the activation site) | site 2 (the inhibition site) | pore state | practically achievable (Yes/No) | defined channel state |
| --- | --- | --- | --- | --- | --- | --- |
| no site occupied | × | × | × | C | Yes | C_0_ |
|  | × | × | × | O | No |  |
| site 0 occupied | √ | × | × | C | Yes | C_1_ |
|  | √ | × | × | O | Yes | O_1_ |
| site 1 occupied | × | √ | × | C | Yes | C_2_ |
|  | × | √ | × | O | No |  |
| site 2 occupied | × | × | √ | C | No |  |
|  | × | × | √ | O | No |  |
| site 0 & site 1 occupied | √ | √ | × | C | Yes | C_3_ |
|  | √ | √ | × | O | Yes | O_2_ |
| site 0 & site 2 occupied | √ | × | √ | C | Yes | C_4_ |
|  | √ | × | √ | O | No |  |
| site 1 & site 2 occupied | × | √ | √ | C | No |  |
|  | × | √ | √ | O | No |  |
| all 3 sites occupied | √ | √ | √ | C | Yes | C_5_ |
|  | √ | √ | √ | O | No |  |

Note: √: site occupied; ×: site unoccupied; C: pore closed; O: pore opened; C_0_, C_1_, C_2_, C_3_, C_4_, and C_5_ represent different closed states of the PAC channel, and O_1_ and O_2_ represent two open states.

We have incorporated this table into the Supplementary information **(Supplementary Table 1)** to clarify the deduction of these channel states, text illustrating this table was also included.

**Point 8:** The experiments presented in figure 2B are described rather unclearly. However, the fact that preincubation with the drug at pH7.3 did not inhibit the current during the exposure to pH 5 could be compatible with a fast washout of the drug as soon as the cell was exposed to the pH5 solution. This experiment should be performed with an ultrafast perfusion system ("concentration jump"), to carefully assess drug washout. The observation that the A321C mutant was inhibited at pH 7.3 is interesting. However, the degree of inhibition observed was rather small. I invite the authors to comment on the magnitude of this effect and consider this point more fully in the interpretation of the results.

**Response:**

Thank you for bringing this to our attention.

1) Our experiments were designed to compare the effects of preincubated C77304 on wt-PAC and PAC/A321C mutant channels. The latter shows basal opening independent of protons, whereas the wt-PAC channels are fully closed at pH7.3. Therefore, if C77304 binds to and inhibits the open channel, the PAC/A321C mutant but not the wt-PAC channel is supposed to be inhibited after 100 µM compound preincubation at pH7.3. These two groups were referred to as ‘wt-PAC, 100 µM/pH7.3’ and ‘PAC/A321C, 100 µM/pH7.3’ in the revised Fig. 2B. The bath solution preincubation groups were used as the experimental control and referred to as ‘wt-PAC, pH7.3’ and ‘PAC/A321C, pH7.3’.

We first measured the currents of these four groups by applying pH5.0 stimulation, denoted as ‘1st pH5.0’ in Fig. 2B. Then, we applied either bath solution (pH7.3) or compound (100 µM/pH7.3) and incubated it with the channel for 3 minutes. Later, we replaced the chamber solutions 3 times to remove residual compound in the perfusion chamber, followed by the second pH5.0 (2nd pH5.0) stimulation. As a result, C77304 preincubation effectively inhibited the currents of PAC/A321C mutant but not the wt-PAC channel, and bath solution preincubation did not affect the currents of both wt-PAC and PAC/A321C channels. These data confirmed that the open-state channels but not the closed channels were inhibited during compound incubation at pH7.3.

We believe that the absence of inhibition in the wt-PAC group is not caused by fast washout of the drug by the second pH5.0 perfusion, for two reasons: i) the currents of the PAC/A321C channels were effectively inhibited by the same treatment, which does not support fast washout of the bound drug; and ii) our data in the revised Fig. 1L showed that the wash off of C77304 from the inhibited channels was very slow.

Moreover, as addressed above in point 5 and the data in the revised Figs. 1E-F, the inhibition of C77304 on PAC channels occurred after channel opening. We also designed another experiment to monitor the time-dependent inhibition of preincubated C77304 on the PAC channels to further support the idea that C77304 inhibited the PAC channel by binding to its opening state, please see the revised Fig. 2D and our response to point 9 for more details.

The reviewer is right that the ultrafast perfusion experiments could address this issue further. Unfortunately, we currently do not have access to such as setup and were not able to do this analysis.

2) The fact that the currents of the PAC/A321C channels are only partially inhibited by a saturated concentration of C77304 (100 µM) in this experiment can be explained as follows. The PAC/A321C channel is gated by protons as well (the revised supplementary Fig. 2B), and only the opening channels were inhibited by C77304 during the pH7.3/100 µM C77304 preincubation treatment. However, pH5.0 treatment could also activate those channels that are in the resting and drug-resistant state at pH7.3, contributing to the observed residual currents stimulated by pH5.0. These data also support the idea that C77304 inhibits the open-state channel.

3) We have revised the text describing these experiments as “The state-dependence of C77304 binding to site 2 was first analyzed using a sequential solution perfusion protocol (Fig. 2B; upper panel). Specifically, PAC currents evoked by two consecutive pH5.0 challenges, separated by a pH7.3 incubation with or without saturating concentrations of C77304, were compared to evaluate any current inhibition at pH7.3. Notably, we performed a triple pH7.3 bath solution replacement before the second pH5.0 pulse to remove the residual compound in the chamber. Our data demonstrates that a saturating concentration (100 µM) of C77304 preincubated with wild-type (wt) PAC channel did not inhibit the currents (Figs. 2B-C), suggesting that the inhibition site 2 was not accessible to C77304 in the channel’s resting state at pH7.3. In contrast, the A321C mutant channel, which showed basal opening at pH7.3[9], was inhibited with this preincubation treatment (Figs. 2B-C). Preincubation of either channels with bath solution (pH7.3 only) did not affect their currents (Figs. 2B-C)” (Page 4, Lines 31-41).

**Point 9:** The data of figure 2D are interesting but puzzling for the following reasons:

(i) at pH 7.3 and in the absence of C77304 (black symbols) there is already a sizable current - is this some kind of leakage? At what membrane potential were the recordings performed?

**Response:** Yes, there is some leak current at pH7.3. The P/4 leak subtraction protocol is no longer applicable when recording the PAC currents, which would result in a small but constant leak current in the recordings (50 – 140 pA) (as reported in previous studies). Nevertheless, we would like to emphasize that it’s not a seal leak as cells with seal resistance lower than Giga ohms were discarded in our experiments; here we recorded the PAC currents at the ramp depolarizations from -70 to +80 mV, and this detail has been incorporated into the revised Fig. 2D legend.

(ii) at pH 7.3 and in the presence of the drug (green symbols) the current seems enhanced - what explanation do the authors offer to explain this result?

**Response:** We would like to explain it as some leak current oscillation after 5 minutes of drug preincubation (100 µM/pH7.3, no pulse). We have shown that the PAC channel is not activated by acutely perfused C77304 at pH7.3 (the revised Fig. 2E);

(iii) when the cells were then exposed to pH 4.6 and still in the presence of C77304, the current is transiently activated (blue symbols), but the level reached by the current is well below the amplitude reached in the absence of the drug (red symbols) - does this imply that the compound acted on the channel at pH 7.3?

**Response:** Yes, the peak current in response to the second pH4.6 stimulation is smaller than the first one. We would like to note that the compound is in close proximity to the channel after 5 minutes of incubation, therefore this difference should be attributed to the very fast inhibition of the currents immediately after the channel opening. In fact, if we assume that C77304 binds with the close-state channel and prevents its opening in response to acid stimulation, then we should not be able to observe this “transient activation” event. We explained this in the revised text as: “Most (~80%) channels might be immediately inhibited post activation, due to close proximity to C77304 and the fast inhibition kinetics”;

(iv) at the end of the experiment (blue symbols) the current amplitude reached values below the current measured at pH 7.3 - what explanation can the authors offer for this observation?

**Response:** 100 µM C77304 quickly and fully inhibits the PAC channels when they are reactivated by the second pH4.6 pulse. We double-checked the data and confirmed that the sudden rundown at the time point of approximately 540 s in the previous Fig. 2D was caused by a leak current oscillation.

Thank you for bringing these to our attention. To further solidify our conclusion, we performed a new batch of experiments with the same protocol, which produced consistent data. We have incorporated these new data into revised Fig. 2D.

**Point 10:** Pg 4, lines 29-30 "Collectively, these data demonstrate that C77304 inhibits the PAC channel by promoting O1-->C4 and O2-->C5 transitions".  It seems that the authors have virtually no evidence to support such a specific statement. As I noted above, the model seems to be based on questionable assumptions. Furthermore, a multistate model is of limited value in the absence of appropriate mathematical reconstruction of the model and simulation of the currents and drug effects. The data presented in the text may simply allow the proposition that C77304 may preferentially bind to a generic open state of the channel (although the data of Fig 2D do not even fully agree with this conclusion).

**Response:**

Thank you for these valuable comments.

In this revision, we have proved that site 0, site 1, and site 2 are indeed located on the PAC channel. Based on these data and a thorough analysis, we proposed a simplified model in Fig. 2A to illustrate channel’s state transitions driven by proton and/or compound binding. These channel states can be inferred from the experimental data. We believe this simple model fully recaptulates the bidirectional modulation of C77304 on the PAC channel. Please see our response to point 7 for details.

Based on this model, C77304 should bind with and inhibit either the proton-activated PAC channel (promoting the O_1_→C_4_ state transition) or the proton and compound-activated PAC channel (promoting the O_2_→C_5_ state transition) (the revised Fig. 2A).

**Point 11:** Pg 4, line 30 "C77304 did not potentiate PAC currents at pH 7.3 (fig 2E)". However, figure 2D shows an increase in the current.

**Response:**

We apologize for the confusion caused by our unclear data presentation. The experiments in Fig. 2E, which tested the effect of acutely perfused C77304 on the PAC channel at pH7.3, do support that C77304 does not directly activate the PAC channel. Regarding the leak current oscillation in Fig. 2D, please see our response to point 9 for details.

**Point 12:** Page 4, line 40 - page 5, line 19. The authors present some interesting data here. However, I do not think the data can conclusively demonstrate that the channel has "an intrinsic proton gating independent voltage gating machinery". To fortify this conclusion, the authors may need to, for example, be able to detect some possible gating currents or demonstrate more fully that the permeating ion may serve as a voltage sensor.

**Response:**

Thank you for this valuable suggestion. The acid evoked Cl^-^ currents mediated by wt-PAC channels in response to a ramp depolarization from -70 mV to +80 mV are outwardly-rectifying, which means that depolarizing voltages increase the channel’s open probability. In the present study, we discovered that there is no endogenous built-in voltage sensor in the PAC channel, and that the rectification direction of the currents through the channel pore is determined by the flow direction of selectively-conducted ions. These data provided evidence for an ion-flux gating model. We designed new experiments to investigate this issue and these data were now incorporated into the revised MS (the revised Figs. 3G-M; the revised Supplementary Figs. 3B-D):

1) We mutated the K319 residue in the PAC channel to E, Q, or H, which changes its charge (positively charged, negatively charged, or neutral) and thus the current direction through the channel pore. We found that both PAC/K319E and PAC/K319Q channels are activated by strong hyperpolarization, whereas the PAC/K319H channel could be activated by both strong depolarization and strong hyperpolarization at neutral pH7.3 (the revised Figs. 3H, 3J, 3K, and 3L; the revised Supplementary Figs. 3B-D).

To confirm that the voltage-activated PAC channel has similar ion selectivity as that activated by protons, we first analyzed the ion selectivity of these channels by an ion substituting strategy. As shown in the revised Figs. 3G-I, the wt-PAC channel conducts outwardly-rectifying Cl^-^ currents, and both the PAC/K319E and PAC/K319Q channels conduct inwardly-rectifying Na^+^ currents, when activated by strong depolarization and strong hyperpolarization at neutral pH7.3, respectively. However, the PAC/K319H channel could conduct both inwardly-rectifying Na^+^ currents at strong hyperpolarization and outwardly-rectifying Cl^-^ currents at strong depolarization, at pH7.3 (please see the revised Figs. 3K-M). We also noticed that the wt-PAC showed certain degree of permability to SO4^2-^, and the PAC/K319H mutant channel showed certain degree of permability to both SO4^2-^ and NMDG^+^, when solely activated by transmembrane voltage at pH7.3.

**The following explanation addresses the conclusion that the PAC channel is voltage-gated by the ion flux.**

2) if the voltage sensor in the PAC channel is made up of channel’s charged residues (a built-in voltage sensor), there are two possibilities:

2-a), assuming that some charged residues, aside from K319, contribute to the voltage sensor in the PAC channel, the PAC/K319E and PAC/K319Q mutant channels harboring the intact voltage sensing machinery like the wt-PAC would be activated by strong depolarization, which contradicts the experimental data (the revised Figs. 3G, 3H, and 3J; the revised Supplementary Figs. 3B-D).

2-b), if K319 makes the voltage sensor, the charge-reverse mutation (K319E) might override the voltage dependence of activation and render the channel activated by hyperpolarizations, whereas neutralizing it (K319Q) is expected to abolish voltage gating. The PAC/K319H channel, however, would be activated either by strong depolarization or strong hyperpolarization, depending on the charge state of the introduced histidine residue, as determined by its pK_a_ value in its local biochemical environment and the external pH. Nevertheless, the PAC/K319H channel would not be activated by both depolarization and hyperpolarization in this scenario. As addressed earlier, the experimental data do not support the assumption that K319 constructs the voltage sensor either (please see revised Figs. 3H, 3J, and 3K; the revised Supplementary Figs. 3C-D).

3) Consequently, we conclude that the PAC channel lacks an inherent voltage sensor. We propose an ion-flux gating mechanism accounting for the proton gating-independent voltage gating of the PAC channel. Specifically, the conducting ion dictates the voltage activation of both the wild-type PAC channel and its mutants, similar to what has been reported for the K2P channel _(Schewe, M. et al., doi:10.1016/j.cell.2016.02.002)_. The experimental data support this mechanism, for which the direction of current rectification is determined by the flow direction of selectively conducted ions, which means the flow ions gate the channel to increase its open probability:

3-a), the wt-PAC channel is selective for Cl^-^, strong depolarizations drive the Cl^-^ ions flow into the cell, which drives the channel open and produces outwardly-rectifying Cl^-^ currents;

3-b), the PAC/K319E and PAC/K319Q mutant channels are selective for Na^+^, strong hyperpolarizations drive the Na^+^ ions flow into the cell, which drives the channel open and produces inwardly-rectifying Na^+^ currents;

3-c), the PAC/K319H mutant channel is selective for both Cl^-^ and Na^+^, strong hyperpolarizations drive Na^+^ ions flow into the cell which drives channel opening and produces inwardly-rectifying Na^+^ currents, while strong depolarizations drive Cl^-^ ion flow into the cell, exhibiting outwardly-rectifying currents.

4) In this paper, we introduce the idea that the PAC channel is gated by voltage independent of its proton gating. We also used the voltage gating to investigate the potential structural role of the candidate proton sensor(s) on the channel. Although the present data support that the ion-flux gating underlies the voltage-dependent gating in the PAC channel, its detailed mechanisms are still to be clarified in future studies. It is likely that the residue constituting the selectivity filter in the wild-type and mutant PAC channels, the 319th lysine(the wt-channel), glutamate(the K319E mutant), glutamine(the K319Q mutant), or histidine(the K319H mutant), acts as the interacting site of the conducting ion to transmit the voltage gating signals.

5) We have incorporated these new data into the revised Fig. 3, and texts related to this figure were also amended (Page 6, Lines 4-43).

**Point 13:** Page 5-6 section entitled: "Mapping key residues involved in the proton gating of PAC channel and uncovering activation site 1". It is unclear how the docking was performed and to which degree the docking exercise was unbiased (i.e. not restricted to a predefined area of the channel). More details also need to be provided on the constraints that were imposed during the "subsequent" docking of C77304 around R237 (pg6, lines 7-8).

**Response:**

Thank you for bringing this to our attention. We have added additional details for molecular docking in the Methods section as:

Molecular docking

The molecular docking was performed as previously described [1, 2]. Rosetta Ligand application from Rosetta program suite version 2019 was used to dock C77304 to PAC channel and Monte Carlo algorithm was employed for sampling [3]. The three-dimensional conformation of C77304 was generated by Frog2 server [4]. The structure of PAC channel (PDB ID: 7JNA) was relaxed using RosettaRelax procedure and the model with lowest score was used for further docking.

Docking included three stages: In the first, low-resolution stage, C77304 was initially placed into the pocket formed by two neighbored subunits in the upper extracellular domain (ECD) [5]. “center of mass” of C77304 was constrained to move within a 10 Å diameter sphere, where it could move freely during the docking process. The second, high-resolution stage employed the Monte Carlo minimization protocol in which the ligand position and orientation were randomly perturbed by a small deviation (0.1 Å and 3°); channel residue side chains were repacked using a rotamer library; the ligand position, orientation, and torsions and protein sidechain torsions were simultaneously optimized using Quasi-Newton minimization and the end result was accepted or rejected based on the Metropolis criterion. The third and final stage was a more stringent gradient-based minimization of the ligand position, orientation, and torsions and the channel torsion angles for both side chains and backbone.

A total of 30000 models were generated by docking and the top 10 models with lowest binding energy were chosen as candidates for further analysis.

References:

[1]	Yang F, Xiao X, Cheng W, Yang W, Yu P, Song Z, Yarov-Yarovoy V, Zheng J. Structural mechanism underlying capsaicin binding and activation of the TRPV1 ion channel [J]. Nat Chem Biol, 2015, 11(7): 518-524.

[2]	Zhang H, Lin J J, Xie Y K, Song X Z, Sun J Y, Zhang B L, Qi Y K, Xu Z Z, Yang F. Structure-guided peptide engineering of a positive allosteric modulator targeting the outer pore of TRPV1 for long-lasting analgesia [J]. Nat Commun, 2023, 14(1): 4.

[3]	Kahraman A, Herzog F, Leitner A, Rosenberger G, Aebersold R, Malmstrom L. Cross-link guided molecular modeling with ROSETTA [J]. PLoS One, 2013, 8(9): e73411.

[4]	Miteva M A, Guyon F, Tuffery P. Frog2: Efficient 3D conformation ensemble generator for small compounds [J]. Nucleic Acids Res, 2010, 38(Web Server issue): W622-627.

[5]	Ruan Z, Osei-Owusu J, Du J, Qiu Z, Lu W. Structures and pH-sensing mechanism of the proton-activated chloride channel [J]. Nature, 2020, 588(7837): 350-354.

**Point 14:** The sections described at page 6 and 7 almost read as a separate paper. In general, I would advise that the current paper focusses on the drug screening exercise (the description of which is completely omitted from the manuscript), followed by a characterisation of the effect of C77304. The data involving characterisation of PAC channels from different species, for example, do not seem to be - strictly speaking - relevant to the study. Furthermore, some statements about gating mechanisms do not seem to be fully supported by the data presented and it may be advisable to have a separate paper devoted to elucidation of these mechanisms.

**Response:**

Thank you for these valuable comments.

In the present study, we uncovered the proton-gating mechanism and introduced the proton gating-independent voltage gating of the PAC channel, based on which we proposed a polymodal gating model of the PAC channel.

We would like to emphasize our rationale for conducting this research is as below:

Pharmacological tools are commonly used as molecular probes to investigate the action mechanisms of ion channels. From a screening campaign, we identified C77304 as a unique bifunctional modulator of the PAC channel, which we believe could be used to explore the channel’s proton gating. Therefore, we first uncovered the action mechanism of the compound and identified the proton-gating associated residues on the channel by using C77304 as the molecular probe.

Mutational analyses are commonly used to investigate the role of specific amino acid residues in the function of proteins. In regard to the proton sensing residues in the proton-gated ion channels (PAC channel in the present study and the acid-sensing ion channels), mutating the primary proton sensing residues (glutamine, aspartate, and histidine) to alanine, eliminates the channels’ proton binding capacity and consequently is supposed to abolish the proton gating. However, in such as case, we cannot discriminate the possibility that loss of function is due to loss of a bona fide proton-binding residue or from a structural perturbation of the channel _(Matthew L. Rook et al., Coupling structure with function in acid-sensing ion channels: challenges in pursuit of proton sensors)_. In the present study, we found that the PAC channels have a unique proton-gating independent voltage gating, which can be used to assess the possible structural role of candidate proton sensing residues. That is, mutating the proton sensing residue to alanine should abolish the proton gating of PAC channel but not affect its proton-gating independent voltage gating. Using this strategy, we confirmed that E181, E257, and E261 likely constitute the proton sensor of the human PAC channel.

Finally, PAC channels are evolutionally conserved, suggesting the proton sensors identified in the human PAC channel should be conserved in PAC channels from other species. Indeed, cross-species analysis (*H. glaber*, *P. textilis*, *N. naja*, *G. gallus*, and *D. rerio*) confirmed this speculation, which further solidified our conclusion. By using molecular dynamics simulations, we also proposed a possible model illustrating the proton gating trajectory of the PAC channel.

Relatively minor comments:
**Point 15:** Abstract, page 2: remove (i) adverbs that imply a personal interpretation of the data (i.e. "excitingly") and (ii) reference to confirmatory data (line 9), which should not be a main outcome of the study.

**Response:** Thank you, we have revised it in this revision.

**Point 16:** Page 2, line 44: "proton sensing mechanism in PAC remains unknown" should read "…remains poorly understood", because recent papers have indeed offered important insights into the gating mechanisms of this channel and it is therefore incorrect to state that these mechanisms are "unknown".

**Response:** Thank you, we have revised it in this revision (Page 3, Lines 2-3 in this revised MS).

**Point 17:** Methods: some important details are missing. These include (i) accession number for each cDNA used in the study and (ii) details of the perfusion system used in the electrophysiology experiments.

**Response:** Thank you for bringing these to our attention. We have added these details.

i) We have added the accession number for each cDNA used in the “Cell culture, plasmids and transient transfection” subsection of “Materials and methods” (Page 13, Lines 14-22);

ii) We have added details for the drug/solution perfusion as “Drugs and solutions were applied by gravity perfusion. Briefly, the solution in the perfusion chamber was removed by a vacuum system connected tubing, and the substituting solutions were applied simultaneously by a perfusion pipette placed near the recording pipette. This operation was repeated for 2 times to ensure complete replacement of the original solution in the chamber in most cases. ” in the “Electrophysiology” subsection of “Materials and methods” (Page 14, Lines 11-15 in this revised MS).

**Point 18:** Page 3, line 14: remove statement "for the first time", because it should be implicit that research presented to a major international journal should be novel and not just a confirmation/repetition of existing knowledge.

**Response:** Thank you, we have deleted it in this revision (Page 3, Line 17 in this revised MS).
