# Supplementary material for: A new polymodal gating model of the proton-activated chloride channel
Source: PLoS Biol. 2023 Sep 15;21(9):e3002309. doi: 10.1371/journal.pbio.3002309 (PMC10529583; doi:10.1371/journal.pbio.3002309)
Supplement: Attachment — Submitted filename: 20230627_PAC MS_point-by-point response letter_TC_Raj.doc [file pbio.3002309.s016.doc]

**point-by-point response to Reviewer#2**
I genuinely appreciate the authors' efforts to answer all my concerns. However, I still have problems with the manuscript.

**Response:**

We greatly appreciate the reviewer’s valuable comments and suggestions, which have greatly improved our manuscript. Below, we address the remaining concerns.

**Major concerns:**
**Point 1:** The work is trying to determine the gating mechanism of PAC channels. This is a significant problem that needs to be experimentally addressed. When dealing with gating, it is necessary to show and analyse data related to gating: macroscopic conductance vs voltage or Po vs voltage or macroscopic conductance vs ligand concentration or Po vs ligand concentration. Neither of these are shown in this work. To make the matter more complicated, PAC has a non-Ohmic behaviour. Thus, its macroscopic outward rectification is caused by a variable single-channel conductance; as the membrane potential became positive, the single-channel conductance increased. Therefore, inferring the gating mechanism from the current-voltage relationships or the dose-response curves alone would be difficult.

**Response:**

Thank you for these comments. We agree with the reviewer that the gating kinetic parameters provide quantitative descriptions of the gating of ion channels (e.g., the EC_50_ (half maximal effective concentration) for ligands activating the ligand-gated ion channels, the V_1/2_ (half maximal activation voltage) of the voltage-gated ion channels, and the relative slope factors which reflect the channel’s sensitivity to changes of ligand concentration and transmembrane voltage].

In the previous version of manuscript, we reported the pH_50_s and the slope factors of protons activating the PAC channel, in the presence and absence of C77304. Regarding the protonation-independent voltage gating of the PAC channel, we have added new experiments analyzing the normalized conductance (G/G_max_) vs. voltage (V) relationship in this revision.

Specifically, PAC channels were clamped at 0 mV, and currents were elicited via a cluster of 2s depolarizations from -100 mV to +200 mV (in 20 mV increment). The macroscopic conductance (G) was calculated using the equation G = I/(V - V_rev_), where I, V, and V_rev_ represents the current amplitude, the depolarizing voltage and the reversal voltage (0 mV), respectively. As G does not reach the maximum at +200 mV, the maximum G (G_max_) cannot be experimentally determined. Instead, it was calculated by fitting the G-V relationship of each recording using the Boltzmann equation: (Y - Y_min_)/(Y_max_ - Y_min_) = 1/(1 + exp[(V_1/2_ - V)/K)]), in which V_1/2_, K, Y_max_, and Y_min_ represent the half maximum activation voltage, slope factor, maximum response, and minimum response, respectively. Here, Y_max_ is the G_max_. The conductance at 0 mV was estimated by the averaged values at -20 mV and +20 mV.

Next, the normalized G (G/G_max_) was plotted as a function of V and the resulting curve was fitted using the Boltzmann equation as well.

The G/G_max_ vs. voltage relationship of PAC channels at pH7.3 was as shown below:

The V_1/2_ and slope factor (K) of the G/G_max_ - V curve was determined as 198.7 ± 13.4 mV and 31.7 ± 3.4 mV, respectively.

We have incorporated these data into the **revised Supplementary Fig. 3B**, text related to this figure was accordingly revised as “As observed from the steady state conductance-voltage relationship, the voltage-dependent activation of PAC channels at pH7.3 illustrated a low voltage sensitivity and incomplete activation even under strong depolarization of +200 mV, with the maximal half activation voltage (V_1/2_) and slope factor (K) being determined as 198.7 ± 13.4 mV and 31.7 ± 3.4 mV, respectively (Supplementary Fig. 3B)”, on page 6, lines 7 - 11, in this revision.

**Point 2.** It is still unclear how the C77304 helped the authors infer the gating mechanism. This compound has a bimodal action, which differs from a bidirectional mechanism. It can enhance the macroscopic current in the middle range concentration and inhibit PAC current at high concentrations. The authors propose that C77304 changes the threshold of PAC to respond to protons. By doing so, it is helping channel gating. It is quite possible, but it still is too blurry how C77304 helps.

**Response:**

Thank you for these comments. Below we explain our rationale for conducting this study:

Pharmacological tools are commonly used as molecular probes to investigate the action mechanisms of ion channels. From a screening campaign, we identified C77304 as a unique bifunctional modulator of the PAC channel, which we believe could be used to explore the channel’s proton gating. Therefore, we first characterized the mechanism of action of the compound and identified the proton-gating associated residues on the channel by using C77304 as the molecular probe. These residues, even though they did not abolish the channel’s proton gating when mutated, form part of the channel’s proton gating machinery. It’s worth noting that C77304 may be used as a pharmacological probe for rapid identification of residues involved in the proton gating of the PAC channel, by a scanning mutation of the full-length PAC channel protein (as discussed on page 11, lines 40-42, in this revised MS).

Mutational analyses are commonly used to investigate the role of specific amino acid residues in the function of proteins. Regarding the proton sensing residues in the proton-gated ion channels (PAC channel in the present study and the acid-sensing ion channels), mutating the primary proton sensing residues (glutamine, aspartate, and histidine) to alanine, eliminates the channel’s proton binding capacity and consequently is expected to abolish proton gating. However, in such a case, we cannot discriminate the possibility that loss of function is due to loss of a bona fide proton-binding residue or from a structural perturbation of the channel _(reference: Matthew L. Rook et al., Coupling structure with function in acid-sensing ion channels: challenges in pursuit of proton sensors_. _J Physiol. 2021;599(2):417-30. Epub 2020/04/20. doi: 10.1113/JP278707. PubMed PMID: 32306405; PubMed Central PMCID: PMCPMC7869053)_. In the present study, we found that PAC channels have a unique protonation-independent voltage gating which can be used to assess possible structural role of candidate proton sensing residues. To put it another way, if we change the specific amino acid that senses protons to alanine, it is highly likely that the proton-controlled gating mechanism of the PAC channel will be eliminated, but the voltage-controlled gating mechanism will remain unaffected. By employing this approach, we have verified that E181, E257, and E261 are the probable components responsible for sensing protons in the human PAC channel.

Finally, PAC channels are evolutionally conserved, suggesting the proton sensors identified in the human PAC channel might be conserved in PAC channels from other species. Indeed, cross-species analysis (*H. glaber, P. textilis, N. naja, G. gallus, and D. rerio*) confirmed this speculation, which further solidified our conclusion. By using molecular dynamics simulations, we also proposed a possible model illustrating the proton gating trajectory of the PAC channel.

However, we acknowledge that the present study did not resolve the question of how C77304 activates the PAC channel (i.e., how the compound binding promotes proton binding with the channel and/or the opening of the protonated channels) – this will be investigated in our future studies.

**Point 3:** The authors proposed three models and claim that model 1 "fully recapitulates the bidirectional modulation of C77304 on the PAC channel". In any case, it is hard to judge the ability of the proposed models to explain the data without fitting the data (G vs V or Po vs V curves; see point 1) with the equations describing the models. Therefore, my suggestion is to move the mechanistic sections to the discussion and leave there as a hypothetical model that may help to explain the data.

**Response:**

Thank you for this suggestion.

We agree to the reviewer’s comments in point 1 that extracellular protons as well as membrane depolarization increase the open probability (P_o_) of the PAC channel, and, possibly the channel’s single channel conductance as well. In fact, it remains unknown if the PAC channel has a uniform single channel conductance at different depolarization voltages, as inferred from the available literature. For example, Wang and Okada et al. reported in 2007 (DOI 10.1007/s00424-006-0193-z) that the single channel conductance of the endogenous PAC channel in HeLa cells is the same at different depolarizing voltages, whereas Lambert and Oberwinkler showed in 2005 that the endogenous PAC channel in HEK293 cells displays a strong single-channel conductance outward rectification (DOI: 10.1113/jphysiol.2005.089888).

If the single channel conductance of the PAC channel remains constant at different depolarizations, the channel’s opening could be simply described as a single state transition from the closed (C) to a uniform opening (O) state as the scheme I shown below:

Scheme I: C→O; in which protons and membrane depolarizations acted to promote the C→O transition;

On the other hand, if the PAC channel has a outwardly-rectified single channel conductance, then multiple open states exist (O_x1_, O_x2_, O_x3_, ……O_xn;_ O represents the open state, and the subscripts (_x1, x2, x3, ……xn_) indicate the channel’s different open state with varied single channel conductance), and the channel’s opening should be illustrated as the following scheme II:

Scheme II: C→O_x1_→O_x2_→O_x3_→……O_xn_; in which protons and membrane depolarizations necessarily act on the C→O_x1_ state transition and possibly the Ox_1_→Ox_2_→Ox_3_→……Ox_n_ transitions.

Nonetheless, both schemes I and II would fit the proposed model in our manuscript (now shown in **Supplementary Fig. 9A**). In this study, we are investigating the mechanisms of protons and depolarization voltages promoting the C→O or C→O_x1_ state transitions (from the closed to the very first opening state). We found that several acidic residues formed the proton sensors and the transmembrane voltage activated the channels using an ion-flux mechanism.

It’s worth noting that the model in the revised Supplementary Fig. 9A is the most pared down model illustrating the state transitions promoted by C77304 and proton binding, in which C77304 and protons necessarily promoted the C→O or C→O_x1_ state transitions, without considering the possibility that C77304 and/or protons promoted the channel transitions between different opening states (O_x1_→O_x2_→O_x3_→……O_xn_). In fact, based on our data, it is rather unlikely that C77304 activated the PAC channel by increasing the channel’s single channel conductance because:

1) C77304 shifted the wt-PAC channels’ threshold activation as well as the half activation pHs to alkaline, suggesting C77304 facilitated proton binding with the channel and/or the opening of the protonated channels (i.e., C77304 increased the channel’s open probability (P_o_) at less acidic pHs);
2) If we hypothesize that C77304 activates the PAC channel by increasing the channel’s single channel conductance, it is expected that low concentrations of C77304 would potentiate proton-activated PAC currents independent of pHs. However, the data showed that C77304, at all concentrations, inhibited but not activated PAC currents at pHs lower than 5.0, which argues strongly against this hypothesis;
3) C77304, at all concentrations, inhibited mutant channels with basal opening probability at pH7.3 (e.g., the PAC/A321C mutant channel), which also provides evidence against the compound activated the channel by increasing its single channel conductance.

We acknowledge the reviewer's suggestion regarding the utilization of single channel recordings to provide direct evidence of the compound's effect on increasing the channel's open probability (P_o_). However, due to the PAC channel's relatively small single channel conductance, conducting such experiments presents significant technical challenges that we were unable to overcome in this study. Additionally, it is important to note that the question of whether the PAC channel exhibits outward rectification in terms of single channel conductance remains unresolved. Moreover, considering the perspective of single channel recordings, it is plausible that the compound could also decrease the channel's P_o_ by binding to the inhibition site 2. This would introduce further complications in the analysis. In summary, while we acknowledge the potential insights that single channel recordings could provide, the technical difficulties associated with the PAC channel's conductance, the unresolved issue of outward rectification, and the possibility of the compound influencing P_o_ through inhibition site 2 make it challenging to include these measurements in our analysis.

Following the reviewer’s suggestion, we have moved the model in Fig. 2A to the discussion section (now shown in supplementary Fig. 9A), using it as a hypothetical model to illustrate our data. The related text was accordingly revised in this revision (page 11, lines 21 - 31).

**Point 4:** The manuscript needs editing. The description of the manuscript is still poor. For example, Figures 1 and 2 are difficult to understand. Punctuation needs revision; it is a particular problem in the Figure legends.

**Response:**

We apologize for the grammatical errors and have now edited the manuscript to improve its clarity. The changes are marked in red in this revision.

**Minor points:**
**Point 1:** The words bi-functional or bi-modal instead of bidirectional would be better descriptors of the effects of C77304 on PAC.

**Response:**

Thanks. Per your suggestion, we have changed bidirectional to bifunctional throughout the manuscript.

**Point 2:** The abstract needs revision to include the conclusion of the C77304 experiments.

**Response：**

We have revised the abstract to add the conclusion of the C77304 experiments: “C77304 acted as a weak activator of the PAC channel, causing moderate activation by acting on its proton gating. However, at higher concentrations, C77304 acted as a weak inhibitor, suppressing channel activity. This dual function was achieved by interacting with two modulatory sites of the channel, each with different affinities and dependencies on the channel's state”, following the suggestions from both reviewers (page 2, lines 4 - 8).

**Point 3:** Abstract line 7 needs editing: Furthermore, we revealed a protonation-independent voltage activation of PAC that appear to follow the ion-flux.

**Response：**

We have revised the text as suggested: “we discovered a protonation-independent voltage activation of the PAC channel that appears to operate through an ion-flux gating mechanism” (page 2, lines 8-10).

**Point 4:** Page 4, line 7: because most of the channels were in the open state.

**Response：**

We have revised the text as suggested: “When the pH was below 5.0, C77304 consistently inhibited PAC currents because most of the channels were in the open state (Fig. 1G)” (page 4, lines 20 - 21).

**Point 5:** Protonation-independent voltage gating of PAC instead of noncanonical proton gating-independent voltage gating.

**Response：**

We have revised the text as suggested (page 5, line 34).

**Point 6:** Page 5, lines 32-33: This is not necessarily true. Lambert and Oberwinkler showed in 2005 that the endogenous PAC channel displayed a strong single-channel conductance outward rectification.

**Response:**

Thank you for bringing this to our attention. We agree to the reviewer’s point. As addressed in point 3, whether the single channel conductance of the PAC channel is uniform at different depolarizing voltages remains unknown. We have revised the text as “PAC currents exhibit an outwardly-rectifying behavior, wherein depolarization leads to an increase in the macroscopic conductance. This property can be explained by two mechanisms: voltage-dependent protonation and voltage gating that is independent of protonation”, on page 5, lines 35-37, in this revision.

**Point 7:** Page 5, line 39: increasing pH

**Response:**

Thanks. We have corrected this error in this revision (page 5, line 44).

**Point 8:** Page 5, lines 41- 42: Figure 2F shows that the current at +40 and +80 mV is nearly 0 when the extracellular pH is set to 6.0. Therefore, the result is not necessarily surprising.

**Point 14:** Page 23, lines 24-25: what exactly do you want to transmit with this sentence?

**Response to points 8 and 14:**

In the previous Fig. 2F (now the revised Fig. 2E), the currents were recorded with ramp depolarizations from -70 mV to +80 mV, which is different from that in Fig. 3E, wherein the currents were recorded with ramp depolarizations from -195 mV to +195 mV.

In PAC channel transfected cells, the acid-evoked currents at +195 mV would easily exceed 50 nA due to a high expression of the channel protein, which would introduce large voltage errors in our recordings. The HEK293T cells endogenously express PAC channels. Consequently, to control for variations in the current amplitude, for experiments in Figs. 3E, we recorded the currents of endogenously expressed PAC channels in HEK293T cells (the acid-evoked currents at + 195 mV was controlled to be less than 20 nA). Due to the limited background expression of PAC channels in untransfected HEK293T cells, the strong depolarization activated PAC currents at pH7.3 were also decreased (mean value of 415 pA), but it’s still significantly higher than that recorded in the HEK293T/PAC^-/-^ cells (mean value of 224 pA). To make the statement clearer, we have revised the text as “Untransfected HEK293T cells endogenously expressing the PAC channel were used for controlling the amplitude of the acid-evoked currents at +195 mV”, on page 24, lines 16-17, in this revision.

**Point 9:** Page 6, line 30: It is hard to rule out the presence/needs of accessory subunits. There are structural examples showing proteins without another protein. For example, the CLC channel's structure did not show Bartin. Hence, at this moment, with the available information, we cannot rule out accessory subunits in PAC channels.

**Response:**

We agree and have deleted this statement in this revision.

**Point 10:** Page 7, line 13: acidic-direction shifted threshold activation pHs. Hard to understand; it needs revision.

**Response:**

Thanks for bringing this to our attention. We have revised the text as “those mutants with threshold activation pHs shifted to more acidic values” in this revision (page 7, lines 22-23).

**Point 11:** Page 9, line 37: Why is this result intriguing?

**Response:**

We described it as intriguing because it is the first evidence showing that the residues making the proton sensors of orthologous PAC channels are conserved across species (page 10, line 6). However, we revised it now as “Interestingly”.

**Point 12:** First line of page 12: mechanism instead of model.

**Response:**

We have changed it to mechanism in this revision (page 12, line 25).

**Point 13:** Since you are not measuring proton binding, remove the words association and dissociation and describe the values of the time constant of the observations.

**Response:**

Thank you for bringing this to our attention. We have revised the figure legend as “Time-course of C77304 activating (K) and inhibiting (L) PAC currents and the corresponding wash-out upon perfusion with compound-free solutions. Compound activation and subsequent current recovery at pH5.8 had time constants of 4.5 ± 1.3 s and 15.9 ± 2.5 s, respectively. At pH 4.6, compound inhibition and subsequent current recovery had time constants of 15.9 ± 2.3 s and 82.7 ± 12.9 s, respectively (n = 6-10)”, on page 23, lines 15-18, in this revision.

**Point 15:** The MD simulations data were gathered at 0 mV. Looking at the IV curves collected at pH 4.0, the size of the current is near 0 at 0 mV, which suggests that the open probability is relatively small. Is it a matter of concern about the mechanistic inferences made with these data?
**Response:**

Our MD simulations of the activated state are based on the reported cryo-EM structure (PDB entry: 7SQF). The article on cryo-EM structure indeed mentioned that they obtained the activated conformation under conditions that favor activation. Notably, the activated state structure was obtained without applying external voltage. Therefore, from our perspective, the MD simulations without voltage applied are sufficient to elucidate the gating mechanism.

**point-by-point response to Reviewer#3**
The authors have provided replies to some of the concerns that were raised. In my opinion, the following points still require the authors’ attention:
**Response:**

We greatly appreciate the reviewer’s valuable comments and suggestions, which have greatly improved our manuscript. Below we address the remaining concerns:

**Point 1:** “We have added details of the screening: “Pharmacological agents interfering with the proton gating of the PAC channel are valuable molecular tools for dissecting its gating mechanism. We screened a compound library (from Selleck Chemicals LLC, Catalog No. L3600) for such agents using manual patch-clamp analysis. Compounds demonstrating >50% inhibition (at 10 µM) of PAC currents in HEK293T cells at pH4.6 were considered as positive ‘hits’ and tested further at a less acidic pH of 5.34 to evaluate any possible pH-dependence of inhibition”, on Page 3, Line 27-32, in this revision.”

Several details will need to be clarified for this part of the study to be suitable for a scientific report. Required information includes, but is not limited to: (i) the rationale at the basis of the selection of the library compound; (ii) the number of compounds that were tested; (iii) the number of positive hits that were detected; (iv) the molecular structure of each individual positive hits and how they compare with C77304; (v) the number of independent experiments and appropriate statistics; (vi) membrane potential at which the recordings were performed etc.

It is also unclear how the C77304 was identified from this analysis given that C77304 does not produce 50% inhibition at 10 uM but only a modest activation (see Fig 1H), while 50% inhibition is the declared threshold for identification of positive hits.

**Response:**

Thank you for bringing this to our attention. We have added these details and revised the text as “Pharmacological agents interfering with the proton gating of the PAC channel are valuable molecular tools for dissecting its gating mechanism. We screened a compound library (from Selleck Chemicals LLC, Catalog No. L3600) for such agents using manual patch-clamp analysis. Specifically, a total of 4208 chemical compounds featuring different core structures and structural diversities from this library were tested (at 10 µM) for their ability to inhibit pH4.6-evoked PAC currents in HEK293T cells (currents recorded by ramp depolarizations from -70 to +80 mV). Compounds demonstrating >50% inhibition of the pH4.6-evoked PAC currents were considered as positive ‘hits’ (3 independent assays per compound), which were further tested at a less acidic pH of 5.34 to evaluate any possible pH-dependence of the inhibition. Four hit molecules, designated compounds 77304, 65841, 62975, and 66386 [C77304, C65841, C62975, and C66386; Figs. 1A (upper panel), Fig. 1B, and supplementary Figs. 1A-C], were identified from this screening campaign. Unexpectedly, C77304 but not the other three (at 10 µM) exhibited activation effects on the pH5.34-evoked PAC currents, identifying it as a unique bifunctional modulator and suggested that it might have a high likelihood of interacting with the channel’s proton gating (Fig. 1C and Supplementary Figs. 1A-C). We thus selected and synthesized C77304 for further investigation (Supplementary Figs. 1D-G)”, on page 3, lines 28-43, in this revision.

Please note that the compound library (Catalog No. L3600) currently available for sale at https://www.selleckchem.com contains only 3010 compounds. It is important to mention that we obtained this compound library several years ago, and at that time, it comprised 4208 compounds.

We have added data of the other three hit molecules to **Supplementary Fig. 1A** (their structures as well as representative currents traces showing their inhibition of pH4.6- and pH5.34-evoked PAC currents), in this revision.

It is worth noting that we conducted the screening analysis at pH4.6. At this condition, 10 µM C77304 did produce >50% inhibition of the pH4.6-evoked PAC currents (please see Figs. 1B and 1I).

**Point 2:** “Yes, the reviewer is right that the C77304 and proton-activated PAC channels at less acidic pHs (pH5.34, pH5.6, and pH5.8) accounted only a small proportion of the total channels available.”

I found the terminology used in the sentence unclear and inaccurate. It is not clear/correct to state that PAC channels at less acidic pHs accounted only for “a small proportion of the total channels available”. It is preferable to state that the open probability of the channel in the pH range mentioned above is rather low. Please ensure accuracy and clarity of the text when referring to this idea.

**Response:**

Thank you for bringing this to our attention. We have revised the text as “It is worth noting that, at pH5.6, the channels activated by both protons and the compound accounted for only a small portion of the total channels that could be activated. This was determined by comparing the currents to those evoked by pH4.6 and normalizing them accordingly (Fig. 1H)”, on page 4, lines 21-24, in this revision. We did this set of experiments in Fig. 1H at both pH5.34 and pH5.6, which revealed similar observations. We apologize for the typo error in the previous Fig. 1H, the pH here should be 5.6 but not 5.34.

**Point 3:** “As addressed in revised Fig. 2, …...”. In the resubmitted version of the manuscript, there are a series of revised figures including Fig 1 E,F and Fig. 2D. The authors are invited to clarify how the revision of the dataset was performed. Were experiments removed and replaced with new recordings? If this was the case, the exact criteria for how experiments were excluded will need to be declared in the methods. For example, the data set of Fig 2 D changes in appearance somewhat significantly compared to the corresponding data set in the previous version of the ms, but the number of independent experiments was apparently only increased by 2-3 (from 6-7 to 9). Was the addition of 2-3 recordings sufficient to significantly impact on the appearance of the data set or were other criteria (e.g. leak subtraction, data selection) used to obtain the next data set?
**Response:**

We apologize for not making it clear. The green dot curve in Fig. 1F in the 1^st^ version of MS was misleading, as it suggested some instability in the compound’s activation effect due to an apparent current run-down. Upon further examination, we found that it was caused by contamination from some leaky currents. These misleading data resulted in the concern that the activation effect of low concentrations of C77304 on PAC currents isn’t stable, which is inconsistent with our observations. We consequently discarded these misleading data and repeated the experiments in Figs. 1E and 1F, and made the revised figures based on the data acquired from the new recordings. Notably, we reached the same conclusions based on these two batches of experiments (please see the previous Figs. 1E-F and the revised Figs. 1E-F).

For the experiments in Fig. 2D, we recorded the PAC currents during a sequence of six perfusion treatments [pH7.3 (1)→pH4.6 (2)→pH7.3 (3)→100 μM C77304/pH7.3, without pulse (4)→pH7.3 (5)→100 μM C77304/pH4.6 (6); number in the bracket indicates the sequence of the treatment; please see the protocol].

After the “100 μM C77304/pH7.3, without pulse (4)” treatment for 5 min, we sometimes see a small background current increment in the subsequent section of “pH7.3 (5)” treatment, when compared with that in the previous “pH7.3 (3)” treatment. This should be attributed to leaky currents which increase following a prolonged (5 min) holding at 0 mV. Nonetheless, the current increase was not significant [the mean currents proportion was 0.086 and 0.062, in the “pH7.3 (5)” and “pH7.3 (3)” section, respectively].

To further solidify our conclusion and to avoid misleading the readers, we performed a new set of experiments for Fig. 2D (5 new cells), removed 3 cells with relatively large change when comparing the “pH7.3 (5)” and “pH7.3 (3)” currents from the old dataset, and made the revised Fig. 2D based on the data from the 4 old cells and 5 new cells. It is worth noting that both these batches of experiments supported the previous conclusion we made in the manuscript (please see the previous Fig. 2D and the revised Fig. 2D).

Following the suggestion from Reviewer#2, we have moved the model in the previous Fig. 2A into the discussion part. Therefore, the previous Fig. 2D is now the revised Fig. 2C.

Below please find the data of the 9 separate experimental cells we used to graph this figure.

**Point 4:** “ …new data into the revised Fig. 1H and rewritten the text as “The proton- and compound-activated channels, however, accounted for a small population of the total available channels (Fig. 1H)” (Page 4, Lines 8-10).”
Fig 1H is missing statistical comparison. It seems essential that this is performed and reported in the figures.
Please also see the comment above about the lack of clarity of the expression “total available channels” in this context.

**Response:**

We have added the statistical comparison in the revised Fig. 1H. Here, we used RM (repeated measure) ONE-WAY ANOVA with post-hoc Dunnett analysis to assess the significant differences, based on the experiment design (increasing concentrations of C77304 was applied to the same recording cell, and a total of 10 cells were tested).

As regard to the expression of “total available channels”, we have revised the text as “It is worth noting that, at pH5.6, the channels activated by both protons and the compound accounted for only a small portion of the total channels that could be activated. This was determined by comparing the currents to those evoked by pH4.6 and normalizing them accordingly (Fig. 1H)”, on page 4, lines 21-24, in this revision.

**Point 5:** This figure (Fig. 1H) clearly shows that C77304 is a low potency agonist (and other data sets in the paper show that the molecule is also a weak inhibitor). This should be conveyed in the abstract for clarity; thus lines 4-6 of abstract should read “C77304 acted as low potency agonist and produced moderate activation of the PAC by acting on its proton gating, while simultaneously inhibiting channel activity at much higher concentrations thus also acting as a low potency inhibitor….”

**Point 6:** I still think Fig 1G is misleading (please see dedicated comment in the feedback provided in relation to the first submission of the ms) and it should be removed from the figure.
**Response to points 5 and 6:**

We apologize for not making it clear. The terms “efficacy” and “potency” were used to describe the modulator’s effect on ion channels. In conditions of less acidic pHs (e.g., pH5.8, pH5.6, and pH5.34), the proton- and compound-activated channels accounted for a small proportion of the total channels that could be activated (Fig. 1H; ≈0.13 in maximum). This is likely because the activation effect of C77304 on the PAC channel relies on the channel’s proton gating, and thus a low proton concentration in less acidic conditions limited the compound’s activation effect. From this point of view, we agree with the reviewer that these findings suggest an apparently low efficacy of the compound.

At pH5.34, pH5.6, and pH5.8, C77304 at concentrations of 5 - 10 µM did potentiate the PAC currents by 1.9 - 5.3 folds (Figs. 1C and 1G), with the apparent EC_50_s (reflecting the apparent potencies of the compound) being determined as 0.6 - 2.9 µM (Fig. 1G). Compared with its activation effect, C77304 exhibited relatively lower potency in inhibiting PAC currents (IC_50_s were 15.5 - 26.9 μM at pHs 5.34, 5.6, and 5.8; Fig. 1I). Considering the dose-response relationships of C77304 against the PAC channel were uniquely bell-shaped at pHs 5.34, 5.6, and 5.8, we would like to keep them in Fig. 1G, as it should be informative for the readers to understand the underlying mechanisms. The data shown in Fig. 1H should be able to eliminate the misunderstanding that C77304 activated PAC channels as robustly as strong acid treatment (e.g., pH4.6 treatment).

Following the reviewer’s suggestion, we have revised the text in abstract as “C77304 acted as a weak activator of the PAC channel, causing moderate activation by acting on its proton gating. However, at higher concentrations, C77304 acted as a weak inhibitor, suppressing channel activity. This dual function was achieved by interacting with two modulatory sites of the channel, each with different affinities and dependencies on the channel's state”, on page 2, lines 4 – 8, in this revision.

**Point 7:** “Additional comments on putative binding sites on the PAC channel”
While the authors provide an interpretation of the data in the context of two separate binding sites, it is appropriate to use the expression “putative binding site” on each occasion these sites are mentioned in the text, since additional direct experimental evidence would be required to unequivocally define the proposed sites of action of C77304. Note that the challenges of separating binding from gating effects have already been largely considered in the field (e.g. doi: 10.1038/sj.bjp.0702164).

**Response:**

We agree to the reviewer’s point that mutation analysis cannot discriminate ‘binding effect’ from ‘gating effect’, and thus have revised the text as ‘putative binding site’ throughout the revised manuscript when referring to ‘binding site’.
