# Supplementary material for: A new polymodal gating model of the proton-activated chloride channel
Source: PLoS Biol. 2023 Sep 15;21(9):e3002309. doi: 10.1371/journal.pbio.3002309 (PMC10529583; doi:10.1371/journal.pbio.3002309)
Supplement: Attachment — Submitted filename: R4_20230807_PAC MS_response to editorial requests.doc [file pbio.3002309.s017.doc]

**Point-by-point responses to editorial requests**

Dear Editor,

Thank you for your kind review of our manuscript. Below please find our point-by-point responses to the editorial requests raised on our revised submission (manuscript ID: PBIOLOGY-D-23-00741R2).

(A) Supplementary files (e.g., excel). Please ensure that all data files are uploaded as 'Supporting Information' and are invariably referred to (in the manuscript, figure legends, and the Description field when uploading your files) using the following format verbatim: S1 Data, S2 Data, etc. Multiple panels of a single or even several figures can be included as multiple sheets in one excel file that is saved using exactly the following convention: S1_Data.xlsx (using an underscore).

Author response:

Thank you for bringing this to our attention. We have included all the individual numerical values underlying the summary data in all of the main text and supplementary figures to a single Excel file named 'S1_Data', in which data for each panel of the figure was put in a single sheet. This supplementary data file was uploaded as 'Supporting Information' in our submission of the revised MS.

(B) Please also ensure that each of the relevant figure legends in your manuscript include information on *WHERE THE UNDERLYING DATA CAN BE FOUND*, and ensure your supplemental data file/s has a legend.

Author response:

Thank you for bringing this to our attention.

1) We have added the statement “The numerical data used in all figures are included in S1_Data” to the ‘data analysis’ subsection of ‘Materials and methods;

2) We have added the **S1_Data** legend as: **“**S1_Data- Excel spreadsheet containing, in separate sheets, the underlying numerical data and statistical analysis for Figure panels 1A (lower panel), 1E(lower panel), 1F(lower panel), 1G, 1H, 1I, 1J, 1K, 1L, 2B, 2C, 2D(lower panel), 2E, 2F, 3A(lower panel), 3D, 3F, 3G(lower panel), 3I, 3J, 3M, 4A, 4B, 4C, 4D, 4E, 4F, 4G, 4H, 5C, 5D, 5E, 5F, 6A, 6B, 6C, 6D, 6E, 6G, 7A, 7B(right panel), 7F, 7G, 7H, S2C, S3A(right panel), S3B(right panel), S3F(right panel), S4B, S4C, S4E, S4G, S4I, S5B, S6C, S6I, S8C, and S9D.

(XLSX)”, in this revision.

These changes can be found on Page 16, Line 24, and Page 29, Lines 2-8, in this revision.

(C) Please ensure that your Data Statement in the submission system accurately describes where your data can be found and is in final format, as it will be published as written there.

Author response:

Thank you for bringing this to our attention. We have added the data availability declaration as: “The authors confirm that all data underlying the findings are fully available without restriction. All relevant data are within the paper and its Supporting Information files” when submitting this revised MS.

(D) Please also provide a blurb which (if accepted) will be included in our weekly and monthly Electronic Table of Contents, sent out to readers of PLOS Biology, and may be used to promote your article in social media. The blurb should be about 30-40 words long and is subject to editorial changes. It should, without exaggeration, entice people to read your manuscript. It should not be redundant with the title and should not contain acronyms or abbreviations. For examples, view our author guidelines: [https://journals.plos.org/plosbiology/s/revising-your-manuscript#loc-blurb](https://journals.plos.org/plosbiology/s/revising-your-manuscript#loc-blurb)

Author response:

Thank you for bringing this to our attention. We suggest putting the blurb as: “Unraveling the mysteries of proton-activated chloride channel gating: Cheng Tang, Piao Zhao, and co-workers uncovered the proton gating and protonation-independent voltage gating mechanisms of the proton-activated chloride channel using its unique bifunctional modulator”.

(E) As you address these items, please take this last chance to review your reference list to ensure that it is complete and correct. If you have cited papers that have been retracted, please include the rationale for doing so in the manuscript text, or remove these references and replace them with relevant current references. Any changes to the reference list should be mentioned in the cover letter that accompanies your revised manuscript.

Author response:

Thank you for bringing this to our attention. We confirmed that the reference list is complete and correct, no cited reference was retracted. Thus, no change was made to the reference list in this revision.

Please be noted that we have revised the text ‘Fig. x’ as ‘Figure x’, and ‘Supplementary Fig. x’ as ‘Figure Sx’ throughout the manuscript, according to the figure citation format of Plos Biology. These changes were highlighted in red in this revised MS.
